# Electrophysiological and Behavioral Correlates of Valence, Arousal and Subjective Significance in the Lexical Decision Task

**DOI:** 10.3389/fnhum.2020.567220

**Published:** 2020-10-07

**Authors:** Kamil K. Imbir, Joanna Duda-Goławska, Maciej Pastwa, Marta Jankowska, Aleksandra Modzelewska, Adam Sobieszek, Jarosław Żygierewicz

**Affiliations:** ^1^Faculty of Psychology, University of Warsaw, Warsaw, Poland; ^2^Biomedical Physics Division, Institute of Experimental Physics, Faculty of Physics, University of Warsaw, Warsaw, Poland

**Keywords:** FN450, LPC, LDT, valence, arousal, subjective significance

## Abstract

The emotional properties of words, such as valence and arousal, influence the way we perceive and process verbal stimuli. Recently, subjective significance was found to be an additional factor describing the activational aspect of emotional reactions, which is vital for the cognitive consequences of emotional stimuli processing. Subjective significance represents the form of mental activation specific to reflective mind processing. The Lexical Decision Task (LDT) is a paradigm allowing the investigation of the involuntary processing of meaning and differentiating this processing from the formal processing of the perceptual features of words. In this study, we wanted to search for the consequences of valence, arousal, and subjective significance for the involuntary processing of verbal stimuli meaning indexed by both behavioral measures (reaction latencies) and electrophysiological measures (Event-Related Potentials: ERPs). We expected subjective significance, as the reflective form of activation, to shorten response latencies in LDT. We also expected subjective significance to modulate the amplitude of the ERP FN400 component, reducing the negative-going deflection of the potential. We expected valence to shape the LPC component amplitude, differentiating between negative and positive valences, since the LPC indexes the meaning processing. Indeed, the results confirmed our expectations and showed that subjective significance is a factor independent from the arousal and valence that shapes the involuntary processing of verbal stimuli, especially the detection of a link between stimulus and meaning indexed by the FN400. Moreover, we found that the LPC amplitude was differentiated by valence level.

## Introduction

The way we reach the understanding of written words is a crucial issue in explaining how language is processed. A lexical decision refers to the human ability to recognize words, and it can be measured in the Lexical Decision Task (LDT) paradigm (Meyer and Schvaneveldt, 1971). This standard task involves a comparison of lexically meaningful words and formally similar pseudowords (Imbir et al., 2015) and a subsequent decision if the presented stimulus comprises a word or not. A pseudoword stimulus has no meaning, however, its construction is consistent with linguistic rules and reflects language’s particularity, such as the use of existing syllables, the possibility to read the word fluently, and adherence to spelling rules (Imbir, 2015). In LDT studies, pseudowords are carefully selected so that they are similar to words in any formal terms, such as length or letter structure. Such stimuli are also used in practice to test the level of proficiency in a foreign language (Lemhöfer and Broersma, 2012).

The LDT procedure measures are based on reaction time and the accuracy of task performance. Even though subjects are not explicitly asked to process the meaning, the involuntary word meaning processing cannot be stopped easily. As a result, there is a remarkable influence of the differences in semantic features on reaction time and accuracy (e.g., Azuma and Van Orden, 1997; McKiernan et al., 2003; Kuchinke et al., 2005). For this reason, the LDT paradigm gives us an insight into the mechanisms of implicit word processing. Furthermore, the use of Event-Related Potentials (ERPs) allows us to examine the stages of word processing. A precise investigation of changes in neural activity during a lexical decision can expand our understanding of the path from the perception of a visual stimulus to the meaning of the stimulus.

### Stages of Word Processing and Their ERP Correlates

The process of recognizing a written word is complex and consists of several stages that have been identified in LDT experiments with healthy subjects (Besner and Coltheart, 1979; Bentin et al., 1999) and patients, for example, with neglect dyslexia (Ellis et al., 1987). The following stages of visual word recognition were deduced: (1) visual encoding of letters, translation of the letter shapes into a sequence of graphemes and orthographic patterns (Bentin et al., 1999), which may also occur as immediate recognizing the word as a single unit based on the visuals (Marshall and Newcombe, 1977; Van Orden et al., 2001) (2) the activation of lexical structures, and (3) an analysis of the word’s meaning (Imbir, 2016a). The process manifests a hierarchical structure, divided into two levels of operations. Initially, sublexical elements are handled, followed by whole-word, lexical processing. This process is reflected in electrophysiological brain activity. The following ERP components were observed: N200 (or EPN in the case of emotional, verbal stimuli processing), FN400, and LPC (or P600).

Research has proven that both faces and strings of letters (words and pseudowords) evoke negative components peaking at 200 ms after the start of the stimuli. However, the N200 intracranial distribution was different for letters and non-orthographic stimuli (Allison et al., 1994). The data obtained suggest that the described difference is associated with the processing of letters. The type and speed of the initial stage of word processing are influenced by “bottom-up” factors referring to, for example, aspects of phonology presentation, and “bottom-down” determinants such as the word’s semantic connotations (Bentin, 1989).

Additionally, research into the Reicher-Wheeler paradigm revealed the existence of words and pseudowords superiority. In the classical Reicher-Wheeler task, a string of letters is briefly presented to subjects and subsequently masked (Reicher, 1969; Wheeler, 1970). Next, participants are forced to decide which of the letters occurred in the displayed string. In the cited research, the choice was narrowed down to two characters. On the behavioral level, it was shown that participants more accurately indicated letters that had initially appeared in masked words (in comparison to pseudowords and baseline) and masked pseudowords (in comparison to baseline). Further analysis revealed the existence of orthographic and familiarity effects on an early P150 component, in which the peak amplitude of P150 was growing for non-words, pseudowords, and words stimuli. Additionally, the amplitude of N200, peaking between 160 and 220 ms, was larger for words than non-words, which reflects the lexicality effect (Coch and Mitra, 2010). In turn, the N300 component also varied across conditions – non-words were related to larger amplitude, particularly in the middle sited anterior to occipital region, whereas pseudowords evoked a stronger response than words at posterior sites over the left hemisphere and across the right one. A similar main effect was observed for the N400 component. What is more, P150 and N400 amplitude were observed in the fluency and reading context (Coch and Mitra, 2010).

To summarize, the following observation can be made—whether a string of letters can be fluently read (as is the case with words and pseudowords) influences early sublexical perceptual processes, as well as later higher-level lexical processes. Further stages of processing are related to ERPs occurring later than 200 ms and refer more to semantic processing than its visual counterpart. The research revealed the existence of association with the N400 component (Kutas and Hillyard, 1980b), the large decrease of ERP potential peaking at 400 ms after the auditory or visual presentation of verbal stimuli. This component is observed in the language decoding process (Lau et al., 2008). Initially, research emphasized postlexical semantic processes, however, other studies have revealed the association between N400 and single words and pseudowords processing when presented in sequences and regulated by semantic priming (Bentin et al., 1993). Nowadays, two paradigms are frequently applied to explain the N400 component. The first is the semantic priming paradigm—this paradigm consists of presenting a related or unrelated word before the target stimuli. The second, the semantic-anomaly paradigm, relies on sentential context—congruous or incongruous words are presented as the continuation of a sentence (Lau et al., 2008). Both paradigms are based on the semantic context, which might be congruent or incongruent with the target word. The research revealed that the incongruent condition observed response has a smaller amplitude in 300–500 ms intervals (Kutas and Hillyard, 1980a; Rugg, 1985). The described modulation, also called the “N400 effect,” also refers to the predictability of the end of sentences. Another component similar to the N400 is FN400, located in the frontal regions (Kutas and Federmeier, 2011). Researchers suggest a relationship with recognition of familiar stimuli (Curran, 2000), and not only words but also geometric figures (Voss and Paller, 2007). However, according to the latest studies, this component is associated more with conceptual priming, and the perceived inconsistency that is difficult for the mind to cope with (Kutas and Federmeier, 2011). For this reason, we may expect the FN400 component to be susceptible to the activation level while performing lexical decisions (Imbir, 2016a).

The last essential component is the Late Positive Complex (LPC), whose amplitude peaks between 500 and 800 ms (Citron, 2012). LPC is located in the posterior area and is associated with deeper processing of the word’s meaning. Similarly to another positive component—P300—it is generally associated with attention orientation and stimulus assessment to respond as required by the task (Citron, 2012). The component is also related to the activation of mental representation (Polich, 2007). The LPC amplitude varies depending on the type of task, and the emotionality of its content (Fischler and Bradley, 2006).

Extant research indicates that emotions can significantly affect the aforementioned processes (Citron, 2012), but in most of the discussed studies, explicit verbal processing was engaged. It is, therefore, vital to investigate their role not only in the explicit but also the implicit lexical processing in LDT.

### Impact of Emotionality of Words on Their Processing

To investigate the implications of emotions for word processing, one has to define emotionality (Kagan, 2010; Imbir, 2015). The emotional quality of stimuli can be seen as a combination of several features. Among them, valence and arousal are typically mentioned as independent dimensions (Osgood et al., 1957; Kagan, 2010), both of which constitute the so-called affective space (Russell, 2003) that is the base for core affect. Valence and arousal have been identified in semantic differential studies (Osgood et al., 1957) and operationalized with the use of the Self-Assessment Manikin (SAM) scale (Lang, 1980), the pictorial scale, that allows subjects to indicate how they felt in a particular moment, without using words. Emotional valence can be defined as the experienced pleasantness or unpleasantness in reaction to external stimuli (Osgood et al., 1957; Kagan, 2010; Moors et al., 2013). Words can be said, according to their emotional valence, to be negative, neutral, or positive. The dimension of arousal expresses the degree to which a particular stimulus elicits an excitation or energy, which may be allocated to specific objects interpreted in an effective way (Russell, 2003). Arousal activates appropriate processes to cope with potentially fatal situations or engage in an appealing interaction with a potential sexual partner (Russell, 2003; Moors et al., 2013; Imbir et al., 2017). Arousing stimuli are evolutionarily important and tend to recruit attentional resources. Arousal, therefore, was found to activate and prioritize the actions crucial for biological survival but disrupting more complex cognitive processes, characteristic to the rational mind (Epstein, 2003a), such as cognitive control, reasoning, or complex decision making (c.f. Imbir, 2016b for a review).

**FIGURE 1:**
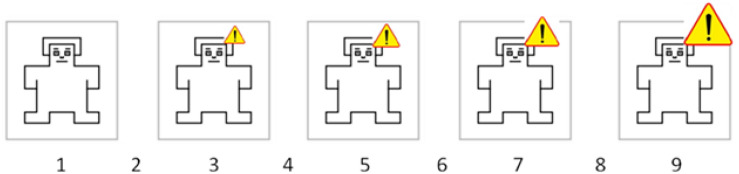
The SAM scale and its description was developed to measure the subjective significance of the affective reaction to verbal stimuli.

With the exception of valence and arousal, another independent emotional dimension called subjective significance was proposed (Imbir, 2016b). Subjective significance can be defined as a reflective form of activation that is analogous to arousal (Imbir, 2015, 2016c). Since arousal activates physiological processes but disrupts high order cognitions, there should exist a mechanism that enables such resource-consuming cognitive processes. Subjective significance can be defined as a feeling or reflective attitude stating that a particular situation is important or crucial for one’s aims and goals, thereby meriting the investment of energy in accurate systematic processing (Imbir, 2016b). This concept can be compared to will-power (Baumeister et al., 1998) or salience (Kahnt and Tobler, 2013). Concerning our goals, certain situations or stimuli are more critical for us than others. We often attach more importance to those which directly relate to us or people who are important to us (Koole and Coenen, 2007). In such situations, we are able to apply systematic thinking, which is more cognitively demanding. Certain circumstances can be different in valence and, at once, equally significant. For example, the win and loss categories have extreme valence loads and are identical in the subjective significance dimension (Imbir, 2016b). Just as arousal is associated with the activation of the experiential mind (Epstein, 2003b; Imbir, 2016b), the subjective significance is the activation mechanism for the reflective system. Each of these three dimensions can influence the implicit word processing, as well as lexical decisions. A useful tool to measure the subjective significance is also the SAM scale (Imbir, 2015, 2016b), consisting of pictograms expressing the importance of the experienced emotional reactions (c.f. Figure 1). The SAM pictures were preceded by the scale’s description aiming to provide an unambiguous interpretation of SAMs. The affective norms for words collected in Polish samples (Imbir, 2015, 2016a) identified the reliability of subjective significance to be high, comparable to arousal, and both dimensions were correlated low; thus, both aspects of emotional activation are notably distinct from each other.

Past studies investigating the effect of valence on LDT performance reported two general patterns of results concerning reaction latencies (Kuperman, 2015), both of which were observed: (1) an inverse-U shape, with faster reaction times for valenced (positive or negative) words compared to neutral words (e.g., Kanske and Kotz, 2007; Schacht and Sommer, 2009), (2) a monotonic decrease in reaction times as valence increases, reported as faster reaction times for positive words compared to negative words (e.g., Hinojosa et al., 2010; Palazova et al., 2013). It is worth noting that emotional words are processed differently from other, more salient emotional stimuli. While processing faces and emotional scenes modulates very early ERP components, emotional word processing has a much more pronounced effect on later ERP components associated with semantic analysis (Palazova et al., 2013). A comprehensive review of EEG and fMRI data (Citron, 2012) reports that the majority of cited studies that employ a LDT show emotional effects (e.g., more negative amplitudes for negative relative to neutral words) starting at a 200–300 ms time-window.

The first of these effects, concerning valence, is called the early posterior negativity (EPN) effect, and is an increase in occipitotemporal negativity for both negatively and positively valenced words relative to neutral words. This effect has been reported in silent word reading (Kissler et al., 2007; Schacht and Sommer, 2009) and lexical decisions (Kanske and Kotz, 2007; Schacht and Sommer, 2009). Valence effects on EPN have been reported to start as early as 100 ms (e.g., Bayer et al., 2012). However, because such early potentials are influenced more by orthographic features, rather than semantic analysis (Nobre et al., 1994), these findings may only reflect conditioned associations with the visual characteristics of valenced, high-frequency words (Schacht et al., 2012).

As for the later N400 component, a study of silent reading by Herbert et al. (2008) reports more negative amplitudes for negative words compared to positive words. This effect was repeated in two LDT experiments by Yao et al. (2016), one using abstract nouns and one using concrete nouns as linguistic materials. However, in an earlier study of lexical decisions, Kanske and Kotz (2007) instead found the amplitude to be more negative for neutral words when compared to valenced words.

The results of our previous study of lexical decisions (Imbir et al., 2016), as well as other studies of ERPs (Curran, 2000; Voss and Paller, 2007; Kutas and Federmeier, 2011), suggest that activity in this time range can be interpreted as the FN400, rather than the N400, component. Namely, our study found more negativity in the 290–375 ms time range for neutral and negative words, relative to positive words, but this effect could only be observed in the frontal regions. This result is congruent with the findings mentioned previously.

Past studies also suggest that valenced words elicit more positive LPC responses during silent reading and lexical decisions, although not in a reliable manner. Some authors (e.g., Cuthbert et al., 2000; Herbert et al., 2006, 2008; Kissler et al., 2007; Schacht et al., 2012) found that the processing of positive words evoked more positive LPC amplitudes than neutral or negative words, while others reported the opposite pattern of results (e.g., Kanske and Kotz, 2007; Hofmann et al., 2009; Schacht and Sommer, 2009; Gootjes et al., 2011). Citron (2012) concludes that these varied results may be due to differences in linguistic materials (nouns vs. adjectives, high-frequency vs. low-frequency words) and tasks.

The effects of arousal on ERPs have not been studied as widely as valence. Citron (2012) reports no conclusive findings on the impact of arousal on the N400 or FN400 components. Some effects of arousal have been observed on the LPC. Delaney-Busch et al. (2016), using a semantic task in which participants had to determine whether each word referred to an animal, showed an increased amplitude of LPC for highly arousing words, relative to words with a low level of arousal. Yao et al. (2016) found a similar effect for lexical decisions to be modulated by concreteness—the effect appeared only in the abstract, but not concrete nouns. Regarding interaction effects, Yao et al. (2016) reported that past results involving an effect of the interaction between valence and arousal on ERPs seem to be inconclusive, citing equally many studies as evidence for and against the existence of such an effect in LDT.

Like valence and arousal, the dimensions of emotional reaction, subjective significance can affect cognitive processes. The subjective significance has been proven to be an independent factor of emotional processing (Imbir, 2016a). An object perceived as significant may be processed differently purely by being meaningful, regardless of other emotional characteristics, e.g., valence. In recent studies, its effect on performance in the Emotional Stroop Task has been observed (Imbir, 2016c). Researchers have also indicated a change in the amplitudes of ERP components (e.g., LPC) at different levels of subjective significance, i.e., self-referentiality of the emotional stimuli correlated to the greater amplitudes of the LPC (Herbert et al., 2011). Nevertheless, a systematic investigation of the impact of valence, arousal, and subjective significance on the implicit processing of meaning in LDT is lacking.

### Aim and Hypothesis

The current study was aimed at exploring the role of valence, arousal, and subjective significance of words in the involuntary processing of meaning in LDT. It is especially vital since emotional load accompanies almost all situations in everyday life. For this reason, we decided to investigate this using a simultaneous, orthogonal manipulation of the three previously discussed factors, because in everyday life the levels of those three factors simultaneously vary across stimuli. This is an important advancement in LDT studies since, in most of the cases, past experiments have been focused on a single factor, or at most two factors at once. We have also made an effort to align the levels of other essential factors such as the frequency of appearance and the length of words that may influence involuntary word processing.

Considering behavioral data, we expected the valence of words to modulate response latency (H1a), either shortening the response latencies to emotional stimuli (negative and positive) in comparison to neutral stimuli, or (H1b) shortening the response latencies to positive stimuli in comparison to negative stimuli. Based on the assumption that subjective significance is the activation mechanism for effortful cognitive processes, we expected (H2) that decisions concerning high subjective significant stimuli should be faster in comparison to low subjective significant stimuli.

Considering the electrophysiological results, we decided to apply two different analytical approaches: the exploratory and the classical, based on components identified in earlier studies. In the exploratory approach, due to the fact that this is the first study investigating valence, arousal, and subjective significance, we predicted (H3) the difference between words and pseudowords to appear on later stages of stimuli processing, later than 240 ms after stimulus presentation, since words and pseudowords share the same core of formal linguistic characteristics. In the classical component analysis, we expected to find correlates of emotional factors in three different components identified in earlier LDT studies to be susceptible to the emotional properties of stimuli: the EPN, FN400, and LPC (c.f. Imbir et al., 2016, LDT). We predicted that (H4) valenced words would elicit a more negative EPN amplitude when compared to neutral words. We also expected (H5) subjective significance to modulate the FN400 component amplitude of ERP. The highly subjectively significant stimuli should reduce the negative-going deflection of potential amplitude in comparison to stimuli with low levels of subjective significance. Finally, we expected (H6) valence to shape the LPC component amplitude, differentiating between negative and positive valences, since the LPC indexes both the processing and the connotations of meaning.

## Materials and Methods

### Participants

The participants were recruited from various faculties of Warsaw universities. They had to meet the following criteria in order to be included in the experimental group: they had to be right-handed native Polish speakers, without chronic clinical issues that may affect EEG recording either directly (e.g., epilepsy) or through medication being taken because of the issue (e.g., anxiety). The participants had either normal or corrected to normal (using glasses) vision. They received a small payment for taking part in the experiment.

The entire experimental group consisted of 36 subjects (18 men and 18 women), aged from 19 to 30 years old (*M* = 21.44; *SD* = 2.82). After collecting the data, some participants were excluded from the EEG analyses either due to artifacts or, because of their extremely short or long response times, they had more than 50% of trials rejected. Effectively, there were 31 participants included in the further analysis, 17 men and 14 women, aged 19–30 years (*M* = 21.39; *SD* = 2.82).

We did not collect any personal data that would allow the identification of the participants. The participants provided written informed consent to participate in the experiment. The design, experimental conditions, and procedure were approved by the Ethical Committee at the Faculty of Psychology, University of Warsaw. All of the procedures involving human participants were done in accordance with the ethical standards of the institutional and national research committee, and with the 1964 Declaration of Helsinki and its later amendments or comparable ethical standards.

### Design

We investigated the behavioral and electrophysiological measures related to the reading of emotional words. We manipulated the factors of valence (3 levels), arousal (3 levels), and subjective significance (3 levels) while controlling the following properties of words: frequency of use in Polish language and length.

### Linguistic Materials

#### Words Selection

Emotional words used in the study were acquired from the Affective Norms for Polish Words Reloaded database (Imbir, 2016a). The database provides eight different affective measures (including valence, arousal, and subjective significance) for 4900 Polish words. In the study establishing said database, each dimension was assessed by 50 participants, half of whom were women, using a SAM scale. The ratings were then converted into means for every scale.

We selected 405 words (nouns), in 27 groups (15 words from each), differing in levels of arousal (low, medium, high), valence (negative, neutral, positive), and subjective significance (low, medium, high), but not in any of the controlled factors. These factors were word length (number of letters) and frequency of use (Kazojć, 2011). Our selection consisted only of nouns, which constitute 59% of the original database. We decided to exclude from the selection all adjectives from the ANPW_R dataset, as, for the adjectives, the biggest obstacle for including them in our study is the fact that polish adjectives possess different forms according to the grammatical gender of the noun they refer to. Possible issues stemming from this fact could not be controlled as the affective norms database we used included only adjectives in their masculine form. It would also mean that each adjective would end on a very similar syllable, making the difference between nouns and adjectives phonologically obvious. The affective norms database used also contains 4 times fewer adjectives than nouns. As our design was complex (3 × 3 × 3), it could present a challenge to pick an equal number of adjectives for each level. Checking the effects of nouns vs. adjectives would further complicate the interpretation of results. The selection of only nouns was also dictated by the need to verify our previous results obtained on lists of nouns.

In our selection, mean ratings for the three levels of arousal were *M* = 3.37, *SD* = 0.38 for words with low arousal, *M* = 3.96, *SD* = 0.21 for moderately arousing words, and *M* = 4.76, *SD* = 0.42 for highly arousing words. As for valence, mean ratings were *M* = 3.96, *SD* = 0.60 for negative, *M* = 5.11, *SD* = 0.31 for neutral, and *M* = 6.13, *SD* = 0.47 for positive stimuli. Likewise, in the dimension of subjective significance, *M* = 3.02, *SD* = 0.31 for low, *M* = 3.62, *SD* = 0.18 for medium, and *M* = 4.35, *SD* = 0.43 for high significance.

To verify our selection, we conducted a 3 (arousal) × 3 (valence) × 3 (subjective significance) ANOVA analysis for each of the five variables (manipulated and controlled), treated as dependent variables. If our selection were valid, we would expect to find only three significant effects, namely an effect of arousal on arousal ratings, of valence on valence ratings, and of significance on significance ratings. The data for the dimension of frequency of use was transformed into natural logarithms to more closely match a normal distribution.

Indeed, we found significant differences between groups divided by arousal in arousal ratings: *F*(2, 378) = 543.95, *p* < 0.01, η^2^ = 0.74, but not in ratings of valence: *F*(2, 378) = 1.40, *p* = 0.25, η^2^ < 0.01, nor subjective significance: *F*(2, 378) = 0.26, *p* = 0.77, η^2^ < 0.01. As for controlled dimensions, there were no differences between groups of different arousal either in terms of word length: *F*(2, 378) = 0.06, *p* = 0.94, η^2^ < 0.01, or frequency of use: *F*(2, 378) = 0.68, *p* = 0.51, η^2^ < 0.01.

For groups divided by valence, we discovered significant differences in valence ratings: *F*(2, 378) = 719.09, *p* < 0.01, η^2^ = 0.79, but not in arousal ratings: *F*(2, 378) = 1.74, *p* = 0.18, η^2^ < 0.01, nor in ratings of subjective significance: *F*(2, 378) = 1.76, *p* = 0.17, η^2^ < 0.01. There were no differences between groups in terms of the number of letters: *F*(2, 378) = 1.07, *p* = 0.34, η^2^ < 0.01, neither were there significant differences in frequency: *F*(2, 378) = 0.65, *p* = 0.52, η^2^ < 0.01.

As regards the groups divided by subjective significance, we found differences in significance ratings: *F*(2, 378) = 570.51, *p* < 0.01, η^2^ = 0.75. The groups did not, however, differ in arousal ratings: *F*(2, 378) = 1.82, *p* = 0.16, η^2^ = 0.01, nor ratings of valence: *F*(2, 378) = 0.51, *p* = 0.60, η^2^ < 0.01. There were also no differences in word length: *F*(2, 378) = 0.06, *p* = 0.94, η^2^ < 0.01, and no differences in frequency of use: *F*(2, 378) = 0.68, *p* = 0.51, η^2^ < 0.01. Furthermore, the analyses showed no significant interaction effects. Specifically, we did not find any of the three possible two-way interactions between manipulated factors (arousal and significance, valence and significance, and valence and arousal) to be significant, either for manipulated or controlled dimensions. We also failed to find a three-way interaction between arousal, valence, and significance on any of the manipulated or controlled scales. All 405 words used in the experiment, as well as their affective measures, length, and frequency, can be found in Supplementary Appendix 1.

#### Pseudowords Selection

Pseudowords were acquired from the Polish Pseudowords List (Imbir et al., 2015), a dataset of 3023 randomly generated pseudowords assessed by competent judges as fulfilling the requirements for pseudoword stimuli. From the list of 1858 words that were given a positive rating by at least four of the five judges, we selected a set of 405 pseudowords that precisely matched the stimuli words in length. To ensure a perfect match, we generated 9 additional 3-letter pseudowords, and so short pseudowords were excluded in the original dataset. The list can be found in Supplementary Appendix 2.

### Procedure

**FIGURE 2:**
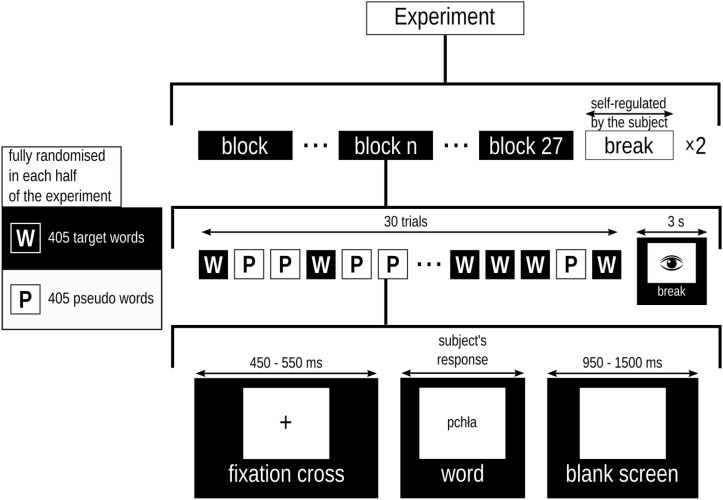
Diagram of the experiment’s protocol.

The subjects sat in a comfortable chair. Words and pseudowords were displayed on an LCD screen at a distance of approximately 1 m from the subjects, in line with subjects’ eyes using size 10 Helvetica font. Participants were encouraged to respond as quickly and as accurately as possible. The task was to read stimuli as they appeared in the middle of the screen and to classify them as words or pseudowords by pressing tagged keys on the keyboard. The response and its latency were recorded. A single experimental block comprised 405 words and 405 pseudowords and was repeated two times. Words and pseudowords were displayed in random order. Trials proceeded as follows: (1) fixation cross displayed for a randomly varied interval between 450 and 550 ms; (2) stimulus displayed until participant responds, but not shorter than for 300 ms; (3) blank screen displayed for a random interval, between 950 and 1050 ms. The experimental protocol provided 3 s breaks for normal blinking every 30 trials. There was one more extended break after 810 trials, for rest and adjustment of posture. The participants controlled its duration. The procedure is outlined in Figure 2.

### EEG Recording

#### Apparatus

The stimuli were displayed on a standard personal computer monitor (LCD display; 17.3-inch diagonal). The stimuli were synchronized to EEG data utilizing a circuit which recorded the changes of the brightness of a small rectangle on display, covered from subjects’ view. Its brightness changed synchronously with the content of the screen. We recorded EEG signal from 19 electrode sites: Fz, Cz, Pz, Fp1/2, F7/8, F3/4, T7/8, C3/4, P7/P8, P3/4, O1/2 referenced to linked earlobes. The ground electrode was placed at the AFz position. All impedances were kept at a similar value below 5 kOhm. The signal was acquired using a Porti7 (TMSI) amplifier, sampled at 1024 Hz.

#### Offline EEG Signal Processing

We conducted the offline signal processing utilizing MATLAB with the EEGLAB toolbox (Delorme and Makeig, 2004) and custom made scripts. The signal was zero-phase filtered. We used the second-order Butterworth filters with 12 dB/octave roll-off, the high-pass filter cut-off was 0.1 Hz, the low-pass cut-off was 30 Hz. Additionally, we used the notch filter for the 49.5–50.5 Hz band, which was also implemented as the second-order Butterworth filter.

We extracted intervals ranging from −200 to 800 ms, with 0 being the onset of the stimulus. The signals were baseline corrected to the interval −200 to 0 ms. We removed from further analysis trials which contained eye blinks, or in which the subject did not correctly identify the color of the presented word. Additionally, we removed trials with response times shorter than the (Q1 – W) or longer than the (Q3 + W) of the logarithm of the response time individually for each subject, where Q1 and Q3 are the first and third quartile, W = 1.5^∗^(Q3 - Q1). Effectively, the response time for the analyzed data across all subjects is within the range of 337.6 ms – 2741.86 ms. The mean number of trials per condition was *M* = 27.3 *SEM* = 0.1.

### Statistical Procedures

The distribution of variables, response accuracy, and the number of correct and artifact-free trials were not Gaussian; therefore, the significance of the effects concerning these variables was assessed using the Kruskal-Wallis rank sum test and Dunn’s test.

The effects concerning other variables, with approximately normal distribution, were assessed using ANOVA with repeated measures in a hierarchical procedure. We investigated behavioral effects (logarithm of reaction time) and the classical EEG component amplitude effects. The significant main effects were analyzed with *post hoc* paired *t*-tests with the Holm’s correction for repeated comparison (Holm, 1979). The significant two-way interactions were similarly investigated using *post hoc* paired *t*-tests with the Holm’s correction. In the case of significant three-way interactions, they were further analyzed by a series of two-way ANOVAs with the levels of a selected variable set iteratively to subsequent levels. The selected variables were permuted. The significance of the effects repeatedly appearing in the series was corrected for multiple comparisons by the Bonferroni correction. The significant two-way interactions were further investigated using *post hoc t*-tests with the Holm’s correction. In case an effect could be obtained by different paths in the hierarchical analysis, we report the most conservative result.

We also performed an exploratory analysis of the EEG effects. In this case, there were additionally two factors that we had to consider: time-window and region of interest (ROI). On the first level of the procedure, we performed a four-way ANOVA with repeated measures—one for each time-window. The significance of the effects repeatedly appearing in the series was corrected for multiple comparisons by the Bonferroni correction. The mean ERP amplitude within a given time-window was the dependent variable, and the independent variables were valence, arousal, significance, and ROI. Similarly, as for the behavioral and classical component-based ERP analyses, we investigated the interaction effects occurring between the factors at subsequent steps through the analysis of variance, which took the interacting factors from a previous step as independent variables. We continued the investigation to a level at which one could understand the interactions in terms of differences in the effects of simple factors, or by the interaction of two factors, under specific conditions determined by the particular levels of the other factors. We performed the *post hoc* using the pairwise *t*-tests. We handled the problem of multiple comparisons by utilizing the Holm procedure. We checked the sphericity with Mauchly’s test and applied the Greenhouse-Geisser correction where necessary. The procedures were implemented in the r statistical package (R Core Team, 2017).

## Results

### Behavioral Results

#### Response Accuracy

The average accuracy for words are not normally distributed, so we compared the medians with the Wilcoxon signed-rank test. Median accuracy for words (*Mdn* = 96.3% *IQR* = 3.3%) and for pseudowords (*Mdn* = 97.8% *IQR* = 3.5%) were significantly different (*V* = 126, *p* = 0.03).

Next, we investigated the accuracy only in reaction to word stimuli. We performed a series of Kruskal-Wallis rank sum tests for each of the design factors. In doing so, we obtained a significant effect of valence [χ^2^(2) = 29.331, *p* < 0.001]. The *post hoc* Dunn’s test showed that the accuracy for positive words (*Mdn* = 98.5%, *IQR* = 2.0%) was higher than for both neutral (*Mdn* = 95.2, *IQR* = 2.8%; *Z* = 4.898, *p* < 0.001) and negative (*Mdn* = 95.6%, *IQR* = 4.3%; *Z* = 4.451, *p* < 0.001) ones. Additionally, the effect of subjective significance was significant in itself [χ^2^(2) = 30.741, *p* < 0.001]. The *post hoc* test revealed that the accuracy increased with the level of subjective significance in the following way: for low level (*Mdn* = 94.1%, *IQR* = 3.0%), for medium (*Mdn* = 96.3%, *IQR* = 4.6%), and for high (*Mdn* = 98.15%, *IQR* = 1.8%). All the pairwise comparisons were significant, i.e., high—low (*Z* = 5.517, *p* < 0.001), high—medium (*Z* = 3.238, *p* < 0.002), and medium—low (*Z* = 2.278, *p* = 0.011). The effect of arousal, on the other hand, was not significant [χ^2^(2) = 2.851, *p* = 0.24].

#### Reaction Time

Reaction times, after the logarithm transform, had an approximately normal distribution, allowing for parametric analysis. The reaction time for words (*M* = 727 ms, *SEM* = 18 ms) was shorter than for pseudowords (*M* = 842 ms, *SEM* = 30 ms), according to the paired *t*-test performed on the natural logarithm transformed values [*t*(30) = −7.045, *p* < 0.001).

**FIGURE 3:**
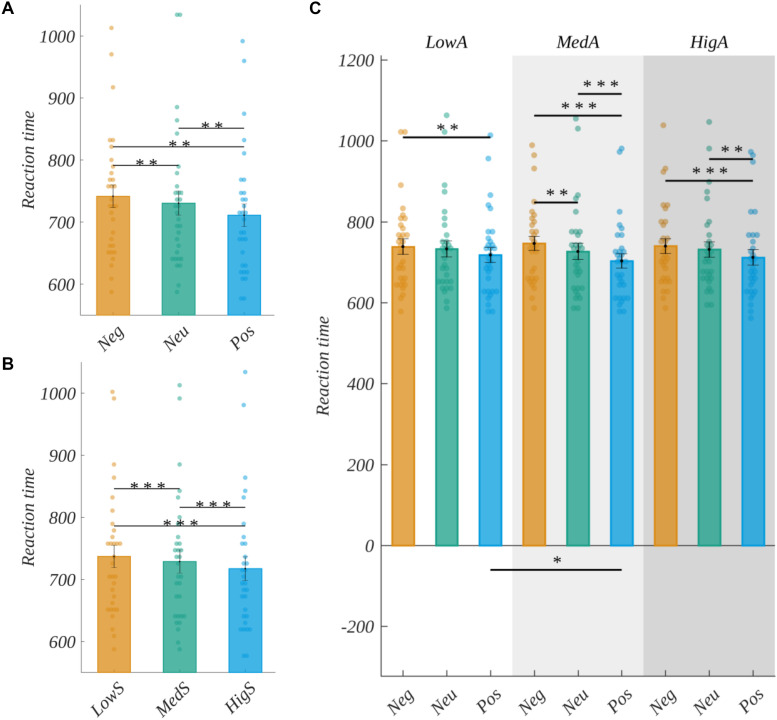
Reaction time for different levels of **(A)** valence, **(B)** subjective significance, and **(C)** interaction between valence and arousal. Average reaction times in msec. Error bars mark *SEM*. Horizontal lines indicate significant differences, asterisks show the significance level (*p < 0.05, **p < 0.01, ***p < 0.001). Circles show results for individual subjects.

In the case of word stimuli, we performed a three-way analysis of variance on the logarithm of reaction time with the valence, arousal, and subjective significance factors. We obtained a significant main effect of valence [*F*(2, 60) = 41.764, *p* < 0.001]. A *post hoc* test showed that the reaction time decreased with the increase of valence level, i.e., for negative stimuli it was significantly longer than for neutral [*t*(30) = 3.407, *p* < 0.002] and then for positive ones [*t*(30) = 8.154, *p* < 0.001]. Moreover, reaction to neutral words was significantly longer than to positive ones [*t*(30) = 6.996, *p* < 0.001]. This relation is presented in Figure 3A.

We also obtained significant main effect of subjective significance [*F*(1.63, 48.93) = 31.852, *p* < 0.001]. Mauchly’s test indicated that the assumption of sphericity had been violated for this factor [χ^2^(2) = 0.774, *p* < 0.024], and therefore degrees of freedom were corrected using Greenhouse-Geisser estimates of sphericity (ε = 0.82). The reaction time decreased with the increase of the subjective significance level, namely the reaction to low significant words was significantly longer than to medium significant [*t*(30) = 4.555, *p* < 0.001], and longer than to highly significant ones [*t*(30) = 6.718, *p* < 0.001]. Finally, for medium significant stimuli the reaction time was longer than for highly significant stimuli [*t*(30) = 4.455, *p* < 0.001]. These properties are presented in Figure 3B, and reaction times for both analyses are reported in Table 1. The main effect of arousal was not significant [*F*(2, 60) = 0.812, *p* > 0.05].

**TABLE 1:** The main effects of valence and subjective significance.

	**Valence**			**Subjective significance**		
	**Negative**	**Neutral**	**Positive**	**Low**	**Medium**	**High**
*M*	742	730	711	737	729	717
*SEM*	18	19	18	18	18	19
*MIN*	584	589	573	588	586	571
*MAX*	1,016	1,033	989	1,001	1,007	1,029

**Mean reaction times are reported, along with minimum and maximum values.**

We observed the significant effect of an interaction between valence and arousal [*F*(4, 120) = 4.702, *p* < 0.001]. For each level of arousal, the reaction time decreased with the increase of the level of valence, but with a different steepness. The interaction is visualized in Figure 3C. *Post hoc* tests showed that, in the case of low arousing words, reaction time was longer to negative than to positive ones [*t*(30) = 4.502, *p* < 0.002]. In the case of medium arousing words, we obtained significant differences between all pairs of valence levels, namely for negative valence the reaction time was longer than for neutral [*t*(30) = 4.099, *p* < 0.007] and for positive stimuli [*t*(30) = 8.616, *p* < 0.001]. Further, it was longer for neutral than for positive stimuli [*t*(30) = 6.051, *p* < 0.001]. In the case of highly arousing words, we observed that the reaction time was longer for the negative than for positive valence [*t*(30) = 5.104, *p* < 0.001]. Moreover, for neutral stimuli it was longer than for positive valence [*t*(30) = 3.980, *p* < 0.009]. Finally, for positively valenced words the reaction time was significantly longer in the case of low arousal, than medium arousal level [*t*(30) = 3.890, *p* < 0.011]. The details of this analysis are reported in Supplementary Appendix 3A (Table 1).

We obtained a three-way interaction effect between valence, subjective significance, and arousal [*F*(8, 240) = 7.378, *p* < 0.001]. To obtain insight into this effect, we proceeded with a series of nine two-way ANOVA tests, each time keeping one level of one of the factors fixed. The detailed results of this analysis are reported in Supplementary Appendix 3B.

### EEG Results

In this section, we report the effects of the manipulation factors on the amplitude of ERP components. The amplitude was measured as the mean amplitude (averaged over the duration of the component) (Luck, 2005) as this is more robust against electrical noise and latency jitter than the maximum amplitude during a given time range. The values of amplitude and the standard error are expressed in μV in the subsequent subsections.

#### Selection of Time-Windows and Regions of Interest

**FIGURE 4:**
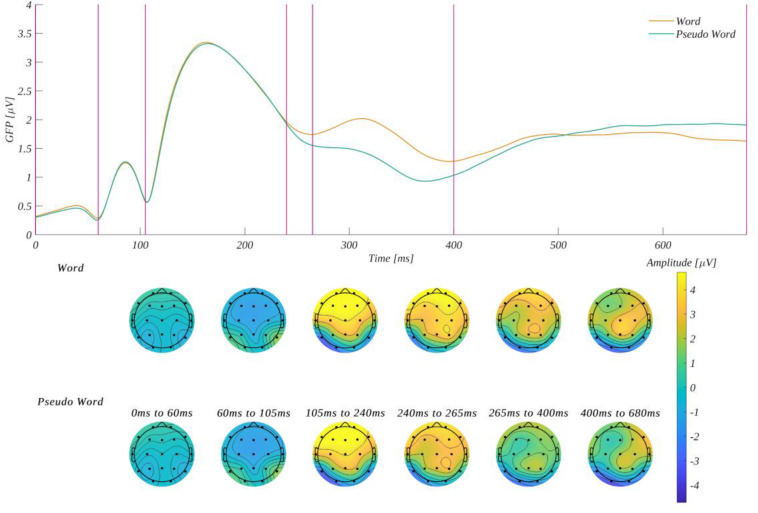
Global field power for words and pseudowords stimuli **(upper part)**, and the topographies of average amplitude in a given time-window **(bottom part)**. The vertical lines in the upper plot indicate time-window boundaries.

ERP amplitude was analyzed in six time-windows: 0–60, 60–105, 105–240, 240–265, 265–400, and 400–680 ms. The selection of these ranges was based on the global field power curve (GFP^1^) for words and pseudowords stimuli (Figure 4). The maxima of GFP for words was used to determine the latencies of the evoked potential components. Microstates corresponding to the components are illustrated in the topographic plots of amplitude distribution at the bottom of Figure 4. An additional time-window, 105–240 ms, was selected to enable the investigation of the first interval when the GFP curve for words deviates from the curve for pseudowords. W selected five regions of interest (ROI): **LF** (Fp1, F7), **CF** (Fz, Cz), **RF** (Fp2, F8), **LP** (C3, P3), and **RP** (C4, P4). In the ROIs, we based our selection on two instances: (1) the ROIs were supposed to be analogical to those selected in our earlier study (Imbir et al. LDT), and (2) the ROIs were supposed to correspond to the classical components identified as being susceptible to the emotional meaning of stimuli in LDT, i.e., EPN, FN400, or LPC.

#### Differences Between ERPs for Words and Pseudowords

**FIGURE 5:**
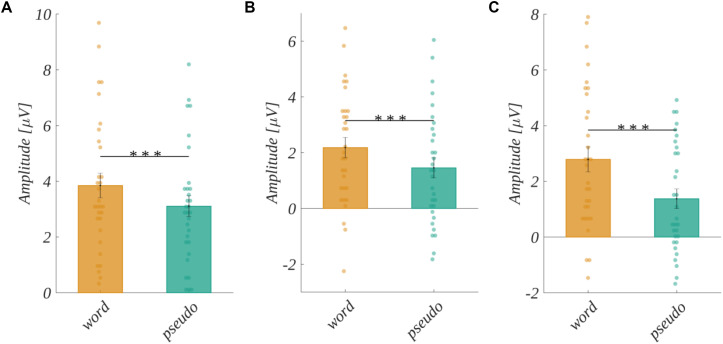
Average ERP amplitudes for words and pseudowords in the following time-windows: **(A)** 240–265 ms; **(B)** 265–400 ms; **(C)** 400–680 ms. Error bars mark SEM. Horizontal lines indicate the significant differences, asterisks show the significance level (***p < 0.001). Circles show results for individual subjects.

The differences between ERPs for words and pseudowords were investigated using a series of two-way ANOVAs with repeated measures with factor stimulus types (word and pseudoword levels) and ROI, one for each time-window. There were no statistically significant results in the first three time-windows, i.e., 0–60, 60–105, and 105–240 ms. In the 240–265 ms time-window, we obtained significant effect of stimulus type [*F*(1, 30) = 35.929, *p* < 0.001]. The amplitude for words was more positive than for pseudowords [*t*(30) = 5.994, *p* < 0.001] (Figure 5A and Table 2). In the next time-window, 265–400 ms, we observed a continuation of this pattern of differences, i.e., there was a significant effect of stimulus type [*F*(1, 30) = 82.707, *p* < 0.001], relying on the fact that the amplitude for words was more positive than for pseudowords [*t*(30) = 9.094, *p* < 0.001 (Figure 5B and Table 2). Moreover, we obtained the interaction between stimulus type and ROI [*F*(2.49, 74.74) = 26.310, *p* < 0.001]. Mauchly’s test indicated that the assumption of sphericity had been violated [(χ^2^(4) = 0.290, *p* < 0.001], and therefore degrees of freedom were corrected using Greenhouse-Geisser estimates of sphericity (ε = 0.62). *Post hoc* tests showed that in all the ROIs the amplitude for words was more positive than for pseudowords, but with different magnitudes. Details of these tests are presented in Supplementary Appendix 3C (Table 1).

**TABLE 2:** Main effects of stimulus type in three time-windows: 240–265, 265–400, and 400–680 ms.

	**240–265 ms**		**265–400 ms**		**400–680 ms**	
	**Word**	**Pseudoword**	**Word**	**Pseudoword**	**Word**	**Pseudoword**
*M*	3.84	3.10	2.79	1.37	2.18	1.45
*SEM*	0.44	0.37	0.45	0.35	0.36	0.36
*MIN*	0.38	0.13	−1.41	−1.78	−2.25	−1.81
*MAX*	9.72	8.13	7.85	4.84	6.53	6.11

In the last time window, 400–680 ms, we again observed the main effect of type [*F*(1, 30) = 24.669, *p* < 0.001]. As in the preceding time-windows, the amplitude for words was more positive than for the pseudowords [*t*(30) = 4.967, *p* < 0.001] (Figure 5C and Table 2). Furthermore, we obtained the interaction between type and ROI [*F*(2.67, 79.97) = 26.510, *p* < 0.001]. Mauchly’s test also indicated that the assumption of sphericity had been violated [χ^2^(4) = 0.299, *p* < 0.001], and therefore degrees of freedom were corrected using Greenhouse-Geisser estimates of sphericity (ε = 0.67). The *post hoc* tests indicated that the amplitude for words was more positive than for pseudowords in three ROI, i.e., CF [*t*(30) = 5.547, *p* < 0.001], LP [*t*(30) = 8.390, *p* < 0.001], and RP [*t*(30) = 4.160, *p* < 0.001] (see Table 3).

**TABLE 3:** The interaction between type and ROI.

	**CF**		**LP**		**RP**	
	**Word**	**Pseudoword**	**Word**	**Pseudoword**	**Word**	**Pseudoword**
*M*	2.54	1.38	1.68	0.27	2.65	1.89
*SEM*	0.54	0.55	0.48	0.46	0.45	0.44
*MIN*	−3.56	−4.81	−3.12	−4.46	−2.33	−1.94
*MAX*	9.08	8.57	6.03	5.38	9.38	7.42

**Time-window 400–680 ms.**

#### Involuntary Word Processing—Exploratory Analysis

The results of the previous subsection showed that the processing of words deviates from that of pseudowords in three time-windows, i.e., 240–265, 265–400, and 400–680 ms. Therefore, we continued the exploratory analysis of ERP effects related to the reading of emotionally laden words in these windows with a series of four-way ANOVAs (ROI × valence × subjective significance × arousal).

There were no significant effects in the 240–265 ms time-window. Specifically, we obtained the following values of statistics for the main effect of valence *F*(2, 60) = 2.24, *p* > 0.05; for the main effect of arousal *F*(2, 60) = 1.89, *p* > 0.05; and for the main effect of subjective significance *F*(2, 60) = 1.17, *p* > 0.05.

**FIGURE 6:**
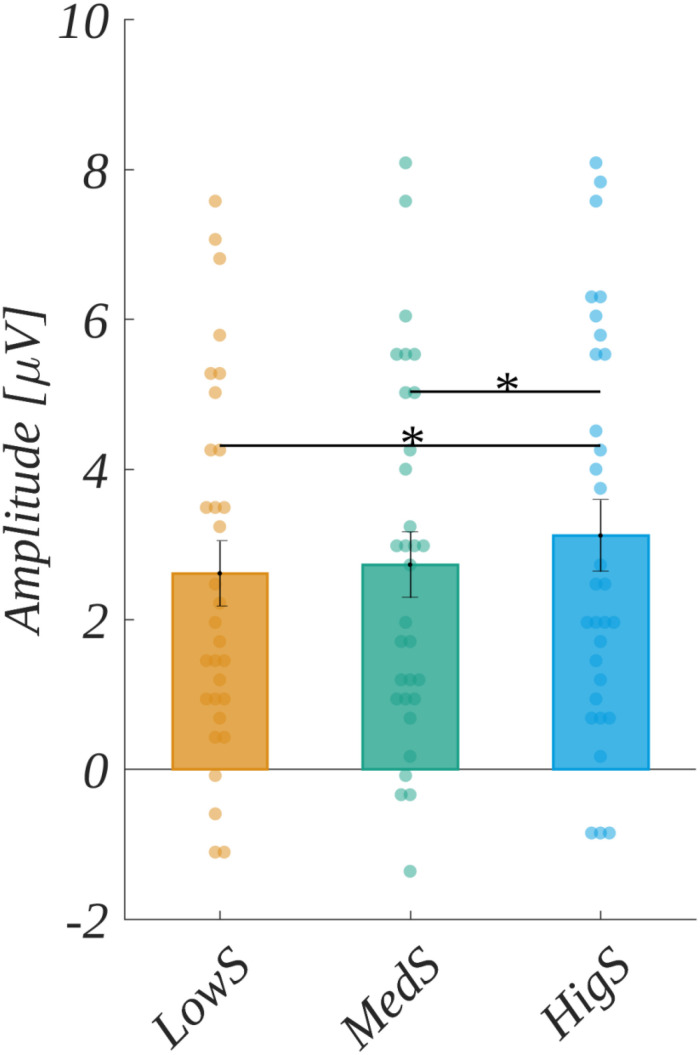
The main effect of subjective significance in the 265–400 ms time-window. Error bars mark *SEM*. Horizontal lines indicate the significant differences, asterisks show the significance level (*p < 0.05). Circles show results for individual subjects.

Next, in the 265–400 ms time-window, the main effect of valence [*F*(2, 60) = 2.32, *p* > 0.05], as well as the main effect of arousal [*F*(2, 60) = 0.43, *p* > 0.05], were not significant. However, we observed a significant main effect of subjective significance [*F*(2, 60) = 8.901, *p* < 0.001]. The *post hoc* tests indicated that the amplitude for highly significant stimuli was more positive than for both medium significant [*t*(30) = 2.895, *p* < 0.014] and low significant ones [*t*(30) = 3.839, *p* < 0.002] (see Figure 6 and Table 4).

**TABLE 4:** The main effect of subjective significance.

	**Subjective significance**		
	**Low**	**Medium**	**High**
*M*	2.61	2.73	3.12
*SEM*	0.43	0.44	0.48
*MIN*	−1.09	−1.44	−0.88
*MAX*	7.69	8.09	8.04

**Time-window 265–400 ms.**

**FIGURE 7:**
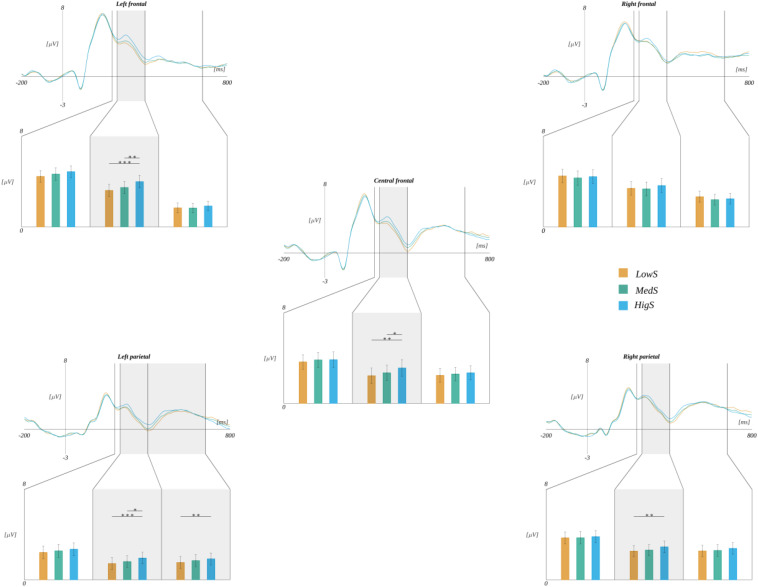
Interaction between regions of interest and subjective significance. In each panel, the upper plot shows grand average ERP traces for each level of subjective significance, whilst the lower bar plot displays the average amplitude within each time-window for each level of subjective significance. Error bars mark *SEM.* Horizontal lines with asterisks mark significant differences (*p < 0.05, **p < 0.01, ***p < 0.001). Time-windows with significant effects are marked with gray shadings.

Moreover, we observed the effect of an interaction between ROI and subjective significance [*F*(8, 240) = 3.570, *p* < 0.001]. Further analyses within each ROI revealed statistically significant main effects related to the levels of subjective significance in the LF region [*F*(2, 60) = 10.339, *p* < 0.001], CF [*F*(2, 60) = 9.646, *p* < 0.001], in addition to LP [*F*(2, 60) = 9.578, *p* < 0.001], and RP [*F*(2, 60) = 5.801, *p* < 0.005]. *Post hoc* tests in these ROIs indicated the following patterns of differences. In the LF region, the amplitude for highly significant stimuli was more positive than for both medium [*t*(30) = 3.202, *p* < 0.006] and low significant ones [*t*(30) = 4.493, *p* < 0.001]. Similarly, in the LP region, the amplitude for highly significant words was more positive than for both medium [*t*(30) = 2.902, *p* < 0.014] and low significant ones [*t*(30) = 4.333, *p* < 0.001]. The same pattern was obtained for the CF region, i.e., the amplitude for highly significant words was more positive than for both medium [*t*(30) = 2.746, *p* < 0.020] and low significant ones [*t*(30) = 3.887, *p* < 0.002]. The pattern of differences in the RP region varied from that described previously. Namely, the amplitude for highly significant stimuli was more positive only from that for low significant ones [*t*(30) = 3.264, *p* < 0.008]. These results are summarized in Figure 7 and Table 5.

**TABLE 5:** The interaction between subjective significance and ROI.

	**Subjective significance**		
	**Low**	**Medium**	**High**
LF	3.26 (0.45)	3.44 (0.47)	3.96 (0.50)
LP	1.37 (0.46)	1.53 (0.46)	1.89 (0.46)
CF	2.42 (0.62)	2.66 (0.61)	3.10 (0.68)
RP	1.89 (0.43)		2.93 (0.45)

**Time-window 265–400 ms. SEM values appear in parentheses below means.**

**FIGURE 8:**
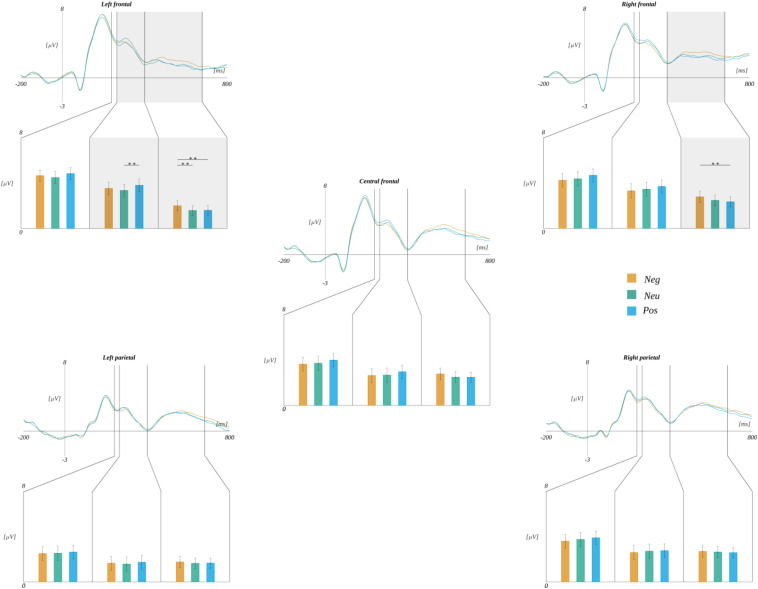
Effects of interaction between regions of interest and valence. In each panel, the upper plot shows grand average ERP traces for each level of valence, lower bar plot displays the average amplitude within each time-window for each level of subjective significance. Error bars mark *SEM.* Horizontal lines with asterisks mark significant differences (**p < 0.01). Time-windows with significant effects are marked with gray shadings.

In the 265–400 ms time-window, we also observed an interaction between ROI and valence [*F*(8, 240) = 3.452, *p* < 0.001]. Further analysis revealed that in the LF region the amplitude for positive words (*M* = 3.77, *SEM* = 0.48) was more positive than for neutral stimuli [*M* = 3.35, *SEM* = 0.47; *t*(30) = 3.418, *p* < 0.006] (cf. Figure 8).

**FIGURE 9:**
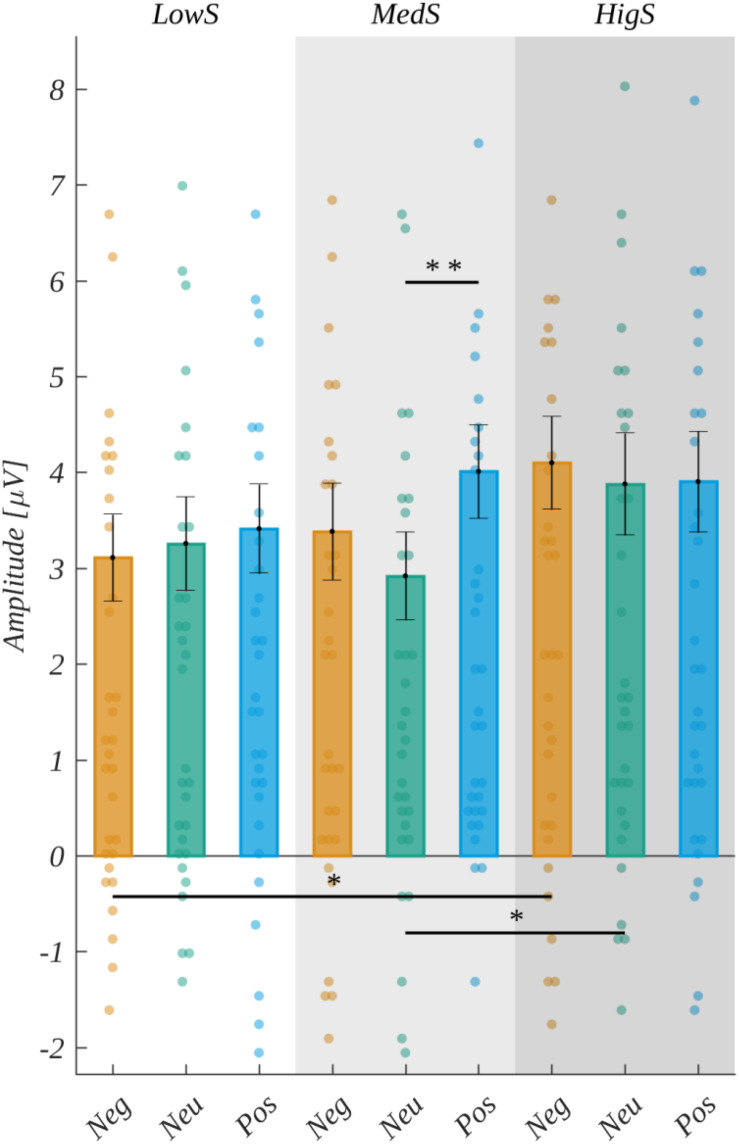
Interaction between valence and significance in the LF region. Average amplitude within the 265–400 ms time-window for each level of subjective significance and valence. Error bars mark *SEM*. Horizontal lines with asterisks mark significant differences (*p < 0.05, **p < 0.01). Circles show results for individual subjects.

Moreover, in the 265 – 400 ms time-window, the effect of an interaction between ROI, valence, and subjective significance [*F*(16, 480) = 2.182, *p* < 0.005] was observed. Further analyses within each ROI revealed a statistically significant effect related to the interaction of valence and subjective significance in the LF region [*F*(4, 120) = 4.317, *p* < 0.003]. *Post hoc* tests showed that it was due to three effects: (i) for negative words the amplitude was more positive in the case of highly significant stimuli (*M* = 4.10, *SEM* = 0.49) than in the case of low significant ones [*M* = 3.11, *SEM* = 0.45; *t*(30) = 3.551, *p* < 0.041], (ii) for neutral words the amplitude was more positive in the case of highly significant stimuli (*M* = 3.88, *SEM* = 0.53) than in the case of medium significant ones [*M* = 2.92, *SEM* = 0.46; *t*(30) = 3.552, *p* < 0.041)] (iii) for medium significant words the amplitude was more positive in the case of positive stimuli (*M* = 4.01, *SEM* = 0.49) than in the case of neutral ones (*M* = 2.92, *SEM* = 0.46; *t*(30) = 4.737, *p* < 0.002). The results for the interaction between valence and subjective significance in the LF region are presented in Figure 9.

In the 400–680 ms time-window, we did not obtain any significant (taking into account the correction for multiple comparisons) simple main effects of valence [*F*(2, 60) = 4.179, *p* < 0.02], arousal [*F*(2, 60) = 4. 039, *p* < 0.023] nor subjective significance [*F*(2, 60) = 2.15, *p* > 0.05.

However, there were statistically significant interactions between ROI and each of the emotional dimensions. Namely, the effect of an interaction between ROI and subjective significance [*F*(8, 240) = 4.497, *p* < 0.001] was observed. Further analyses within each ROI revealed a statistically significant main effect related to the levels of subjective significance in the LP region [*F*(2, 60) = 5.670, *p* < 0.006]. *Post hoc* tests showed that the amplitude related to highly significant words (*M* = 1.90, *SEM* = 0.47) was more positive than in the case of low significant ones [*M* = 1.52, *SEM* = 0.48; *t*(30) = 4.029, *p* < 0.001] (cf., Figure 7).

Furthermore, we obtained an interaction between ROI and valence [*F*(8, 240) = 2.534, *p* < 0.012]. The analyses within each ROI indicated main effects related to the levels of valence in the LF region [*F*(2, 60) = 7.181, *p* < 0.002]. *Post hoc* tests showed that the amplitude related to the negative words was more positive than to both neutral [*t*(30) = 3.222, *p* < 0.006] and positive ones [*t*(30) = 3.528, *p* < 0.004]. A similar effect was observed in the RF region [*F*(2, 60) = 5.394, *p* < 0.007]. Here, the *post hoc* tests showed that the amplitude in the negative condition was more positive than in the positive one [*t*(30) = 3.431, *p* < 0.005] (cf., Figure 8 and Table 6).

**TABLE 6:** The interaction between valence and ROI.

	**Valence**		
	**Negative**	**Neutral**	**Positive**
LF	1.94 (0.35)	1.55 (0.35)	1.52 (0.33)
RF	2.71 (0.44)		2.28 (0.39)

**Time-window 400–680 ms. SEM values are reported in brackets. SEM values appear in parentheses below means.**

**FIGURE 10:**
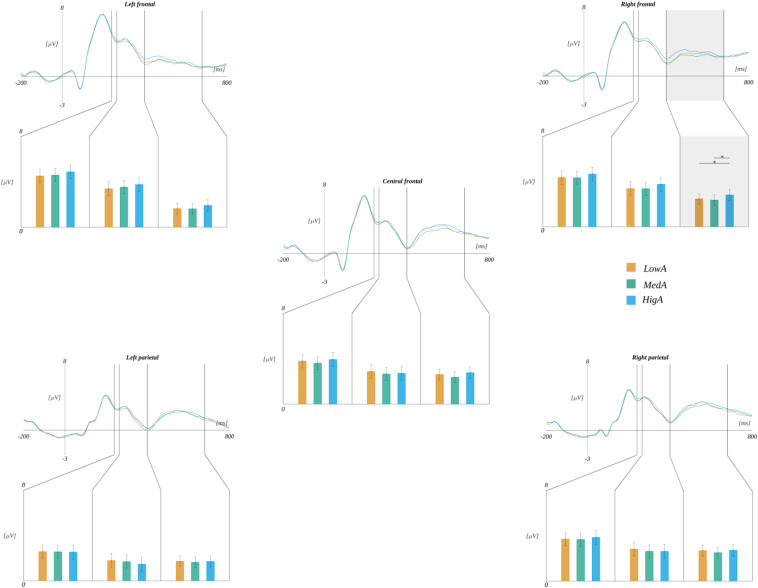
Interaction between regions and arousal. In each panel, the upper plot shows grand average ERP traces for each level of arousal, whilst the lower bar plot displays the average amplitude within each time-window for each level of subjective significance. Error bars mark *SEM*. Horizontal lines with asterisks mark significant differences (*p < 0.05). Time-windows with significant effects are marked with gray shadings.

Finally, we obtained the significant interaction between ROI and arousal levels [*F*(8, 240) = 2.422, *p* < 0.016]. A further analysis within each ROI revealed a simple effect related to the levels of arousal [*F*(2, 60) = 6.324, *p* < 0.003] in the RF region. The *post hoc* test indicated that the amplitude was more positive for highly arousing words (*M* = 2.73, *SEM* = 0.43) than for both medium [*M* = 2.31, *SEM* = 0.39; *t*(30) = 2.982, *p* < 0.017] and low arousing ones [*M* = 2.40, *SEM* = 0.40; *t*(30) = 2.621, *p* < 0.027] (cf., Figure 10).

#### Involuntary Word Processing—Classical Analytical Approach

Besides the exploratory analysis, we investigated the dependence of the amplitude of components known from the literature on the factors studied here. We found statistically significant effects for both FN400 and LPC components and describe the details of these effects below.

##### The EPN component

**FIGURE 11:**
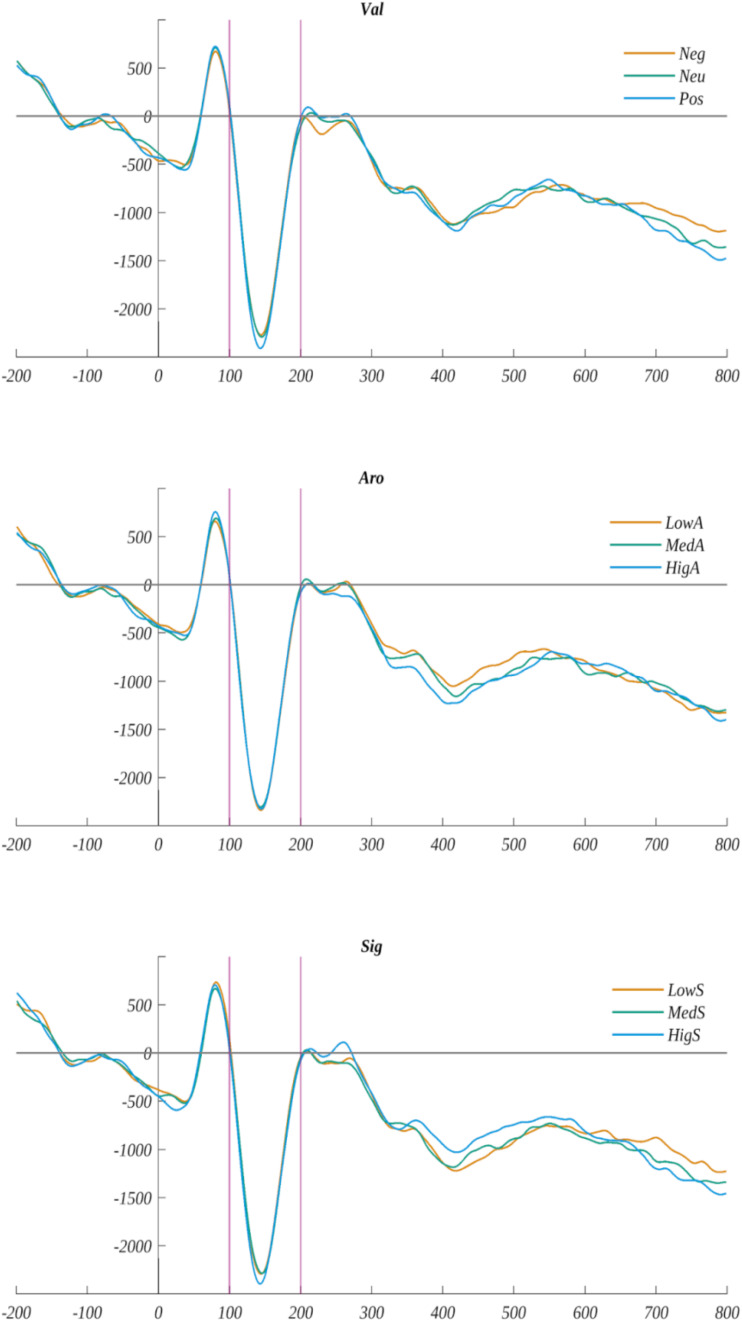
The time course of ERP averaged across electrodes from ROI_*EPN*_ for each level of valence, arousal, and subjective significance. The vertical lines indicate time-window boundaries of the EPN component.

We analyzed ERP amplitude in the time-window: 100–200 ms in the ROI characteristic for the EPN component (ROI_*EPN*_) (i.e., O1, O2, T5, T6). The average signal from ROI_*EPN*_ electrodes for each level of valence, arousal, and subjective significance are illustrated in Figure 11.

In the ANOVA with repeated measures with the average EPN amplitude as the dependant variable and valence, arousal, and subjective significance as the independent variables, we did not identify any statistically significant effects. The test results for the main effects were as follows: for valence *F*(2, 60) = 0.453, *p* > 0.05; for arousal *F*(2, 60) = 0.54, *p* > 0.05; and for subjective significance *F*(2, 60) = 0.144, *p* > 0.05. The interaction effects were not significant either.

##### The FN400 Component

**FIGURE 12:**
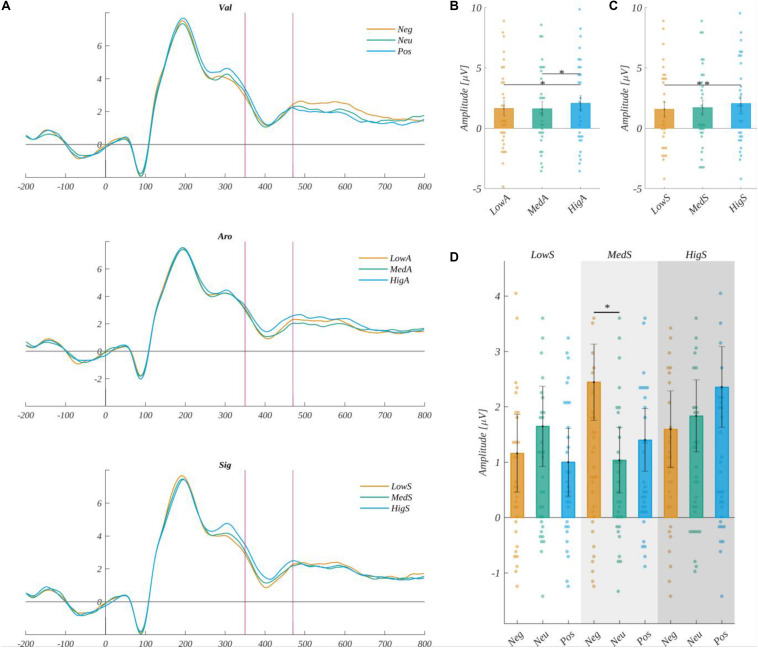
Left: **(A)** The time course of the ERP averaged across electrodes from ROI_*FN400*_ for each level of valence, arousal, and subjective significance. The vertical lines indicate time-window boundaries of the FN400 component. Right: The main effects on amplitude for the FN400 component of: **(B)** arousal, **(C)** subjective significance, and **(D)** interaction between valence and subjective significance for medium arousal. Error bars mark *SEM*. Horizontal lines with asterisks mark significant differences (*p < 0.05, **p < 0.01). Circles show results for individual subjects.

We analyzed ERP amplitude in the time-window: 350–470 ms in the ROI characteristic for the FN400 component (ROI_*FN400*_), i.e., Fp1, Fp2, F3, Fz, and F4. The average signal from ROI_*FN400*_ electrodes for each level of valence, arousal, and subjective significance are shown in Figure 12A).

We obtained a significant main effect of arousal [*F*(2, 60) = 5.595, *p* < 0.006]. The amplitude for highly arousing words was more positive both from low arousing [*t*(30) = 2.546, *p* < 0.033] and medium arousing [*t*(30) = −2.972, *p* < 0.017] stimuli (Figure 12B and Table 7).

**TABLE 7:** Main effects of arousal and subjective significance on the FN400 component.

	**Arousal**			**Subjective significance**	
	**Low**	**Medium**	**High**	**Low**	**High**
*M*	1.64	1.61	2.06	1.57	2.04
*SEM*	0.62	0.60	0.64	0.61	0.63
*MIN*	−4.73	−3.58	−3.62	−4.32	−4.28
*MAX*	9.00	8.47	9.93	8.97	9.45

We also observed the main effect of subjective significance [*F*(2, 60) = 6.124, *p* < 0.004]. The *post hoc* analysis showed that the amplitude for highly significant words was more positive than for words of low subjective significance [*t*(30) = 3.252, *p* < 0.008) (Figure 12C and Table 7). The main effect of valence was not significant [*F*(2, 60) = 0.35, *p* > 0.05].

Moreover, in the case of the FN400 component amplitude we obtained the significant effect of a three-way interaction between valence, arousal, and subjective significance [*F*(8, 240) = 2.627, *p* < 0.009. We performed a further two-way analysis of variance within each level of valence, subjective significance, and arousal. The only effect that, using *post hoc* tests, could be interpreted as a difference in the levels of one factor while keeping the other two constants was that the amplitude for medium arousing, medium significant words that were negatively valenced was more positive than for neutrally valenced ones [*t*(30) = 3.890, *p* < 0.019] (Figure 12D and Table 8). Other differences contributing to this interaction involved changes in the level of more than one factor and so are difficult to interpret.

**TABLE 8:** The main effect of valence on the LPC component.

	**Valence**	
	**Negative**	**Positive**
*M*	2.15	1.85
*SEM*	0.45	0.45
*MIN*	−2.99	−3.33
*MAX*	7.31	6.84

##### The LPC Component

**FIGURE 13:**
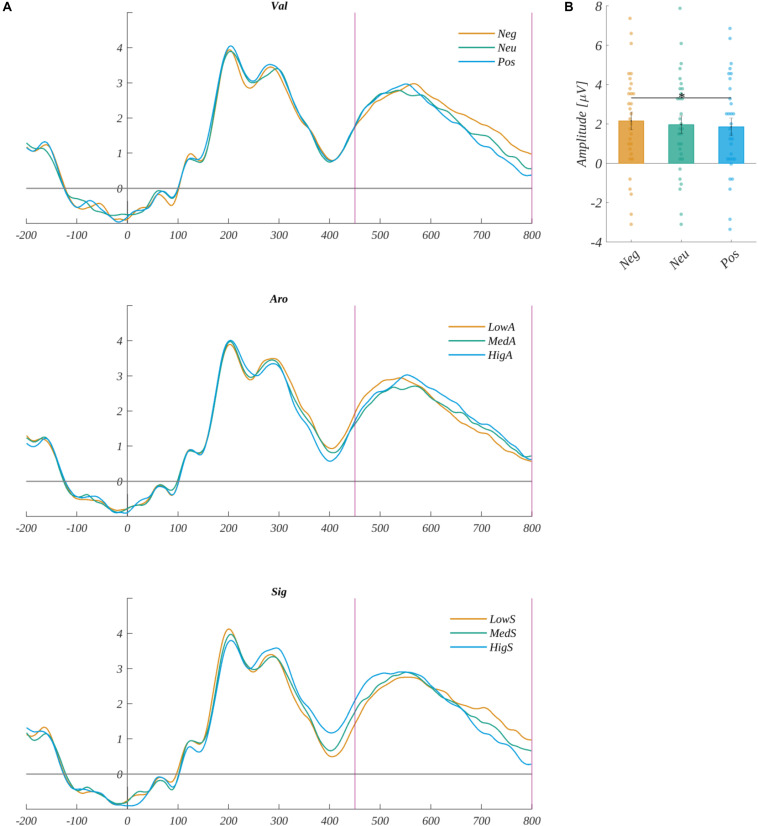
**(A)** The time course of ERP averaged across electrodes from ROI_*LPC*_ for each level of valence, arousal, and subjective significance. The vertical lines indicate time-window boundaries of the LPC component. **(B)** The main effects of valence for LPC component. Error bars mark *SEM*. Horizontal lines with asterisks mark significant differences (*p < 0.05). Circles show results for individual subjects.

The amplitude of the LPC component was analyzed in the 450–800 ms time-window in the ROI (ROI_*LPC*_) characteristic for this component: P3, Pz, and P4. Average signals from the ROI_*LPC*_ are shown in Figure 13A) separate for each level of valence, arousal, and subjective significance. We obtained the main effect of valence [*F*(2, 60) = 3.910, *p* = 0.025]. The *post hoc* tests revealed that the amplitude of LPC was more positive in the case of the negative (*M* = 2.15, *SEM* = 0.45) than the positive valence [*M* = 1.85, *SEM* = 0.45; *t*(30) = 2.576, *p* = 0.045] (cf., Figure 13B). The main effects of arousal [*F*(2, 60) = 0.58, *p* < 0.56] and of subjective significance [*F*(2, 60) = 0.19, *p* < 0.83] were not significant.

## Discussion

The LDT paradigm is a unique research tool for the investigation of the verbal stimuli processing stages, regarding both visual and comprehension levels. The processing of meaning in LDT is implicit and involuntary. What is more, in the context of the task, it can be easily differentiated from the processing of visual features of stimuli. For this reason, LDT can provide us with an insight into emotional word processing and the role of valence, arousal, and subjective significance; factors that facilitate the description of emotional reactions to words.

### Behavioral Results

On the behavioral level of analysis, the dependent variables were the accuracy of responses and reaction time, measured both for words vs. pseudowords comparisons, as well as in comparisons between emotional categories of words. Considering the words vs. pseudowords comparison, words processing accuracy was significantly lower for words (*Mdn* = 96%) compared to pseudowords (*Mdn* = 98%). The difference might be explained by the predominance of less frequently occurring words in the sample used. The subject could incorrectly report the occurrence of pseudowords instead of words when the word was not very familiar for the subject. However, observed differences imply having marginal significance for overall results and might be treated as a ceiling effect resulting from the applied methodology—the number of repeated trials. Considering reaction latencies, generally, reactions for words (*M* = 727 ms) were faster than for pseudowords (*M* = 842 ms). Research proved that words are processed more rapidly than pseudowords (Rossmeissl and Theios, 1982; Liu et al., 2004). The difference can be explained by the theory of reading, which assumes simultaneous and parallel processing of letters in words and relatively direct access and searches for phonological memory (Rossmeissl and Theios, 1982). Words are processed easier, because they have representation in phonological lexicon, which does not occur in the case of pseudowords.

Considering the comparisons between emotional categories of words, we observed a higher accuracy for positive words compared to both neutral and negative words. The valence effect in the accuracy of the identification of words stimuli was previously not directly discussed in our hypothesis. Similarly, the effect of subjective significance on words was detected, as accuracy increased with the level of subjective significance. As previously noted, highly subjectively significant words tend to activate reflective processing; therefore, more effort is exerted, and fewer errors appear. Moreover, an analogous effect of subjective significance was also reflected in electrophysiological results, the amplitude in ERPs and FN400 components for highly significant stimuli was more positive, the component revealed lower intensity (please see the subsequent chapter).

The second dependent variable analyzed for comparisons between emotional categories was reaction time. We observed the negative trend for reaction latencies for valence. The reaction latencies were found to be the longest for negative and the shortest for positively valenced words. This result entirely confirmed the H1a and H1b hypotheses. The obtained results pattern is partially consistent with the current literature. Research showed that faster processing in the LDT paradigm appears for emotionally involved materials in comparison to neutral ones (Kanske and Kotz, 2007; Citron et al., 2014), thus emotionally laden stimuli evoke greater intentional allocation of attention and, consequently, faster processing compared to the processing of the neutral stimuli (Kuperman, 2015). Interestingly, previous research held in LDT paradigm revealed that the functional relation between valence of stimulus and reaction time results in an inverse U-shape (Kousta et al., 2009; Yap and Seow, 2014). However, the evidence of an inverse U-shape is not unquestionable, as there are also reports confirming a linear relation between the described variables (e.g., Hinojosa et al., 2010; Palazova et al., 2013). Research showed that reaction time for positive words was shorter than for negative ones. Our study also confirmed this pattern. A possible explanation of such a pattern might be that the perceptual system is adjusted to track potential danger, thus threatening stimuli capture one’s attention stronger than others (Fox et al., 2001; Ohman and Mineka, 2001). Therefore, negative words distract the subject from the task and completing the task takes longer.

Subsequently, we observed the decrease of reaction time with the increase of subjective significance level. The obtained result confirmed our second hypothesis (H2). Subjective significance is a form of reflective activation, promoting effortful, intentional processing. When the subject perceives a stimulus as important, he invests greater energy in systematic processing (Imbir, 2016b), which in turn results in shorter response latency. Surprisingly, there is highly limited data on the subjective significance role in LTD task performance, which might prove that the current study adequately fills the gap in current knowledge and brings additional value to understanding the role of emotions in the LDT paradigm.

### Electrophysiological Results

At the electrophysiological level, we analyzed the amplitude of ERP in two approaches: the exploratory based on whole signal analysis and in the classical one based on components and localizations typically reported as susceptible to lexical processing. In the exploratory based approach, we were able to identify effects that would be omitted if we kept only to analyze classic components. Nevertheless, many of the differences observed in the exploratory approach directly correspond to the classic components. In this section, we try to take a holistic look at the processes taking place during the task, interpreting both exploratory found and classic effects in the broader context of neuropsychological knowledge following the processes on the timeline from the beginning to end of an ERP.

The first issue is the difference between the potentials recorded for words and pseudowords. The period when ERP for both types of stimuli is the same indicates the formal stage of processing of verbal stimuli. During the period of significant differences in the course of ERP, one may expect an analysis of the meaning of words. Given the exploratory approach, the GFP and ERP timeline shows that the differences between word processing and pseudowords start at around 240 ms after the stimulus onset. This is relatively late for the division between signals for words and strings of letters (Bentin et al., 1999; Coch and Mitra, 2010). This is likely also the main reason why we did not find any results within the EPN component—it occurs before the separation of the signals observed in the current study. The difference between ERPs evoked by words and pseudowords has the same character from the moment of the separation at 240 ms into stimuli processing, up to the last analyzed time-window at 400–680 ms—the amplitude evoked by words is the largest among all the three time-windows. For the first time-window (240–265 ms), the ERP heading to a negative peak (local minimum of amplitude that can be seen on Figures 7, 8) could be interpreted as a late N200 component, despite the fact that the amplitude is positive and that the N200 typically peaks about 50 ms earlier (Schmitt et al., 2000; Folstein and Van Petten, 2007). As this component is related to recognizing familiarity, this effect clearly shows how the participants in this particular moment recognized the words, which were familiar. Nevertheless, the amplitude in the discussed time-window is positive, not negative, and thus the paradigm used likely does not engage many mental activities typically associated with N200 (Schmitt et al., 2000; Folstein and Van Petten, 2007). Despite the negativity of this exploratory-found component, the difference between the signals evoked by words and pseudowords is in line with findings from other studies (Allison et al., 1994; Coch and Mitra, 2010). The process of familiarity recognition could be observed in the following parts of the signal, which could be interpreted as FN400, a component also related to the processing of familiarity (Curran, 2000; Voss and Paller, 2007). Since pseudowords do not have any particular meaning, they therefore do not evoke large amplitudes in later stages of processing, as can be seen in the lower part of Figure 4. We can conclude that the participants did not have any interest in further processing a string of letters after realizing the stimuli is meaningless. On the other hand, the later effect (starting at 400 ms into stimuli processing) could also be interpreted as LPC, as the potentials evoked by words and pseudowords are heading toward positive values (c.f. Figures 7, 8 for illustration). This component is also related to responding to the task, while subjects in these late stages of processing initiated the responses (Citron et al., 2013). Nevertheless, the difference between the signal for words and pseudowords is congruent with the results of previous studies regarding words as specific stimuli (Lau et al., 2008; Coch and Mitra, 2010), as well as those regarding words as generally familiar stimuli (Kutas and Hillyard, 1980a; Rugg, 1985; Curran, 2000; Voss and Paller, 2007).

The second issue surrounding the exploratory analysis was the emotionality of the words used. It appeared that we did not observe any significant effects of emotions in the time-window, where the GFP for words just separated from that for pseudowords and amplitudes for words were found to be significantly more positive than amplitudes for pseudowords (c.f. Figure 5). This is particularly interesting, as this time-window lasted only 25 ms (from 240 to 265 ms into stimuli processing), which can be labeled as the exact moment of division between the signals for both types of stimuli. This part of the signal, to some extent, could be tied to the N200 component (Coch and Mitra, 2010). The task given to the participants to decide whether the presented string of letters is a word or not seem to be so involving up to this moment that emotional properties do not play a role in processing it. Only after realizing the word has actual meaning does the reader start processing the emotional factors.

The first exploratory window picked for the analyses, where we observed the influence of emotional factors on ERPs, starts at 265 ms into stimuli processing, which is an early stage of analyzing the meaning of a word (Bentin et al., 1999). In this time-window, we observed the main effect of subjective significance over the whole scalp besides the right frontal region. The words with high subjective significance evoked the most positive amplitudes. In this early time-window, we also observed the effect of valence in the left frontal region, and the positive words in particular evoke more positive amplitudes than the neutral ones in LF ROI. These differences could also be observed within the group of words with medium subjective significance. We can conclude that at the beginning of processing the emotional factors, the two effects we observed are, in fact, the beginning of processes taking place within the next, i.e., the FN400 component. Highly significant words, evoking higher amplitudes (thus less intense negative deflection on ERP, c.f. Figures 8, 12) within this component could promote more effortful, but also more effective processing, along with positive words—this reasoning seems to be supported by our behavioral results, as both the highly significant and the positive words promoted faster and more effective responses. It is also in line with previous findings regarding the influence of valence on N400 (Herbert et al., 2008; Yao et al., 2016) or FN400 (Imbir et al., 2016), as they also show more positive amplitudes for positive words.

The FN400 classic component started within the time of the first exploratory component described previously. This component measures amplitudes purely from frontal parts of the skull, and it is associated with the detection of a link between stimulus and meaning (Kutas and Federmeier, 2011). For this component, we observe the effect of significance, which has the same pattern of differences as the effect which begun right after realizing the meaning of the word, particularly highly subjectively significant words that evoke the most positive amplitudes. Since FN400 is a negative-going wave, the results can be interpreted as the lower intensity of the FN400 component for highly subjectively significant stimuli, thus the lower level of cognitive conflict experienced by participants when assessing this category of stimuli. Since it has been frequently proven that emotional factors play a role in this component (Curran, 2000; Voss and Paller, 2007; Kutas and Federmeier, 2011; Imbir, 2016c, 2017), we can conclude that subjective significance is another important emotional factor modulating the amplitude of the component. We can see that this process lasts into the next exploratory window (400–680 ms into stimuli processing), and is observed in the left parietal part of the skull (c.f. Figure 7), having the same shape of differences, i.e., higher amplitudes related to highly significant words. It seems to be the same effect, lasting quite a long time due to the structure of the task and late separation between word and pseudoword stimuli. We can conclude that this effect of significance, beginning at the early stage, lasts throughout the large part of word processing. The high subjective significance could promote more effortful processing, but also more efficient, which is supported by the behavioral data—highly significant words are also the ones to which responses were faster and more accurate.

In the FN400 classical component, we also observed the main effect of arousal, in particular highly arousing words evoked higher amplitudes than low or medium arousing ones. This effect is congruent both with studies that operationalized arousal in a similar way (Citron, 2012), as well as other studies, thus outlining the influence of emotionality on this component (Curran, 2000; Voss and Paller, 2007; Kutas and Federmeier, 2011; Imbir, 2016c, 2017). This effect also lasts into the second exploratory time-window picked by us (Figure 10), where it has precisely the same pattern of differences, being observed in the right frontal parts of the scalp—this may also be the same effect observed in two time-windows, as their times overlap. Analogously to the subjective significance, we can conclude that the high load of arousal promotes effortful processing. It is particularly interesting that the effect of arousal was observed only in the right frontal (Figure 10) parts of the skull, which could suggest that certain regions process the emotional properties related to this factor. This reasoning is supported by previous studies reporting arousal processing in the right frontal region (Nitschke et al., 1999; Stoléru et al., 1999; Karama et al., 2002; Dickenson et al., 2019).

In the frontal component of FN400, we also observed an interaction of all three factors manipulated in this experiment, which seems to be challenging to interpret. However, when we explore the interaction, we can see that the difference concerns only mildly arousing, mildly significant words. In this small group of stimuli, negative words evoked higher amplitudes than neutral ones. It seems to be the beginning of a more substantial effect, which is observed in the frontal ROIs in the last exploratory-picked time-window because it has a similar pattern of differences, i.e., negative words evoking higher amplitudes than positive ones (in the right front) or higher than both positive and neutral (in the left front). Due to the specificity of the task and the difference between words and pseudowords occurring late in the time course of the trial, we can conclude that the effect observed in the exploratory time-window is, in fact, the further part of the FN400 potential. The same shape of results (higher amplitudes for negative words) has been reported in previous studies (Herbert et al., 2008; Imbir, 2016c; Yao et al., 2016).

The same shape of results regarding valence lasts into the last classic component picked for the analyses—the LPC, parietal component, where negative words evoke higher amplitudes than positive words. It gives good evidence that in the later parts of processing stimuli valence plays an important role, and brings another voice to the discussion, whether it is the direction of valence that influences the LPC, or purely the valent–non-valent difference (Cuthbert et al., 2000; Kanske and Kotz, 2007; Kissler et al., 2007; Herbert et al., 2006, 2008; Hofmann et al., 2009; Schacht and Sommer, 2009; Gootjes et al., 2011; Citron, 2012; Schacht et al., 2012). The influence of valence, lasting throughout different time-windows, is also reflected in the behavioral results. The negative words evoked more positive amplitudes, but the accuracy of the answers is smaller for the negative stimuli; the reaction times are also significantly slower. We can conclude that the cognitive load generated by negative valence lowers the efficiency in the task.

### Limitations

The study has its limitations. The first group applies to the verbal stimuli used. Due to the focus on three dimensions of emotion, i.e., valence, arousal, and subjective significance, there remains a space for further research on other possible factors in the studied paradigm. Verbal material varies in terms of the levels of each dimension of stimuli. In the case of valence, the division for three levels is indisputable. The question is whether it is possible to identify analogous levels for arousal and subjective significance. However, specifying the medium level gives a reference point for the others—low and high levels of variables. It provides a more detailed insight into the examined matter.

Additionally, the three dimensions utilized in orthogonal manipulation, because those dimensions are correlated to each other, as was revealed in the normative study for words (i.e., Imbir, 2016a), resulted in a selection of stimuli that were rather moderate in intensity. The stimuli that are more intense in negativity or positivity are together more arousing, while neutral stimuli are low in their arousal. This can be observed in three-way interactions when most of the effects can be seen in neutral valence condition. The advantage of such an approach is the unique chance of investigating the mutual impact of all dimensions together on performance in LDT.

As for our EEG data, the reported lack of EPN effects can also be considered as a limitation, explained by the characteristic negative-going deflection occurring before the appropriate time-window for semantic analysis, which may itself reflect a limitation, posed by our use of a mastoid references set-up. This interpretation has been proposed, for example, by Delaney-Busch et al. (2016), who pointed to a comprehensive review of 40 years of ERP findings on affective picture processing (Olofsson et al., 2008). These authors found that reliable emotional effects on EPN had been observed primarily in studies that employ whole-brain average reference electrodes. The data obtained from mastoid references may demonstrate some affective modulation in the appropriate 200–300 ms time range but exhibit different signal characteristics.

## Conclusion

This study is the first showing that subjective significance, together with arousal and valence, influences the involuntary processing of verbal stimuli meaning observed in the LDT paradigm. We have demonstrated that the activational components of emotional reactions (subjective significance and arousal) shape the processing of familiarity or decoding of the link between stimulus and meaning indexed by the FN400. In contrast, valence shapes the post-semantic processing at the LPC component. We have also shown, due to the three-level manipulation, that interactions between valence and subjective significance are present at the FN400 component.

## Data Availability Statement

The raw data supporting the conclusions of this article will be made available by the authors, without undue reservation, to any qualified researcher.

## Ethics Statement

The studies involving human participants were reviewed and approved by the Ethical Committee at the Faculty of Psychology, University of Warsaw. The patients/participants provided their written informed consent to participate in this study.

## Author Contributions

KI: theoretical proposition. KI, MP, AS, MJ, and AM: introduction and results discussion. KI and JŻ: design. KI, JD-G, and MP: method (words). JŻ and JD-G: method (EEG measures) and experimental procedure programming. JŻ, JD-G, and MP: experiment execution. JŻ, KI, JD-G, AS, and MP: statistical analyses. JŻ, JD-G, MP, and AS: results description. JD-G, JŻ, and KI: figures. All authors contributed to final version of the manuscript.

## Conflict of Interest

The authors declare that the research was conducted in the absence of any commercial or financial relationships that could be construed as a potential conflict of interest.

## Footnotes

1 GFP corresponds to spatial standard deviation at a given time. It quantifies the sum of electrical activity over all the electrodes (Lehmann and Skrandies, 1980; Skrandies, 1990).
