# Supplementary material for: Electrophysiological and Behavioral Correlates of Valence, Arousal and Subjective Significance in the Lexical Decision Task
Source: Front Hum Neurosci. 2020 Oct 7;14:567220. doi: 10.3389/fnhum.2020.567220 (PMC7575925; doi:10.3389/fnhum.2020.567220)
Supplement: Supplementary file 3 [file Table_3.DOCX]

## **Appendix 3**

### **Appendix 3A. Details of the analysis of the reaction time two-way interaction between levels of valence and arousal.**

Table A1. Mean reaction time for each level of valence and arousal, along with minimum and maximum values.

|  | Arousal | | | | | | | | |
| --- | --- | --- | --- | --- | --- | --- | --- | --- | --- |
|  | Low | | | Medium | | | High | | |
|  | *M (SEM)* | *MIN* | *MAX* | *M (SEM)* | *MIN* | *MAX* | *M (SEM)* | *MIN* | *MAX* |
| Valence |  |  |  |  |  |  |  |  |  |
| Negative | 739 (19) | 577 | 1021 | 746 (18) | 587 | 987 | 740 (18) | 588 | 1041 |
| Neutral | 733 (20) | 586 | 1059 | 727 (20) | 587 | 1056 | 732 (19) | 591 | 971 |
| Positive | 718 (19) | 581 | 1013 | 703 (18) | 576 | 983 | 712 (19) | 561 | 971 |

### **Appendix 3B. Details of the analysis of the reaction time three-way interaction between levels of valence, arousal, and significance.**

First, we investigated the two-way interaction between significance and arousal within each level of valence. This interaction was significant for negative (*F*(4,120) = 4.451, *p*< .002), neutral (*F*(4, 120) = 6.094, *p* < .001) and positive (*F*(4,120) = 4.086, *p* =.004) valence of the stimuli. Further post-hoc tests within each level of valence yielded the following results. In the case of negative and medium arousing words, the reaction time for high levels of significance (*M* = 718 ms, *SEM* = 18 ms) was shorter than for both medium significance (*M* = 752 ms, *SEM* = 17 ms; *t*(30) = -5.117, *p* < .001) and low significance (*M* = 770, *SEM* = 21 ms; *t*(30) = -5.052, *p* < .001). Moreover, for negative and low significant stimuli the reaction time was longer for medium arousal (*M* = 770 ms, *SEM* = 21 ms) than for low arousal levels (*M* = 741 ms, *SEM* = 19 ms ; *t*(30) = 3.625, *p* < .034).

In the case of neutral and low arousing stimuli, the reaction time for high significance levels (*M* = 701 ms, *SEM* = 20 ms) was shorter than both medium (*M* = 744 ms, *SEM* = 25; *t*(30) = -4.878, *p* < .001) and low significance levels (*M* = 755 ms, *SEM* = 18 ms; *t*(30) = -4.734, *p* < .002).

For neutral and highly arousing words, the reaction time for high significance levels (*M* = 718 ms, *SEM* = 19 ms ) was shorter than for low significance levels (*M* = 751 ms, *SEM* = 20 ms; *t*(30) = -3.977, *p* < .013). Furthermore, for neutral and low significant stimuli the reaction time was shorter for medium arousal (*M* = 724 ms, *SEM* = 20 ms) than high arousal levels (*t*(30) = -4.475, *p* < .003).

Within positive valence, the effect resulted from the interaction between conditions differing simultaneously in their arousal and significance levels, therefore they are not reported here since they are difficult to interpret. The results for interaction between subjective significance and arousal within each level of valence are presented in Fig. A1, top row.

Secondly, we investigated the two-way interaction between valence and significance within each level of arousal. This interaction was significant within low (*F*(4, 120) = 5.740, *p* < .001), medium (*F*(4, 120) = 5.570, *p* < .001), and high (*F*(4, 120) = 5.247, *p* < .001) levels of the arousal factor. Further post-hoc tests within each level of arousal yielded the following results.

For low arousing and medium significant words, the reaction time was longer for negative (*M* = 740 ms, *SEM* = 20 ms) than for positive valence (*M* = 705 ms, *SEM* = 17 ms ; *t*(30) = -3.425, *p* < .049). In the case of low arousing and highly significant stimuli, it was longer for negative (*M* = 735 ms, *SEM* = 21 ms) than for neutral valence (*M* = 701 ms, *SEM* = 20 ms; *t*(30) = 4.214, *p* < .006).

Analysis within medium arousing and low significance words showed that the reaction time was longer for negative (*M* = 770 ms, *SEM* = 21 ms) than in both neutral (*M* = 724 ms, *SEM* = 20 ms; *t*(30) = 6.117, *p* < .001) and positive valence (*M* = 705 ms, *SEM* = 17 ms; *t*(30) = 6.632, *p* < .001).

In the case of medium arousing and medium significant stimuli, the reaction time was longer for negative (*M* = 752 ms, *SEM* = 17 ms) than for positive valence (*M* = 704 ms, *SEM* = 18 ms; *t*(30) = 5.664, *p* < .001). Furthermore, for neutral valence words (*M* = 728 ms, *SEM* = 20 ms) it was longer than for positive ones (*t*(30) = 3.502, *p* < .034).

The analysis within highly arousing and low significant words indicated that the reaction time for positive (*M* = 706 ms, *SEM* = 18 ms) ones is shorter than for both negative (*M* = 751 ms, *SEM* = 19 ms; *t*(30) = -6.219, *p* < .001) and neutral ones (*M* = 751 ms, *SEM* = 20 ms; *t*(30) = -5.601, *p* < .001).

Additionally, within highly arousing and highly significant words the reaction time for positive (*M* = 703 ms, *SEM* = 21 ms) was significantly shorter than for negative valence (*M* = 736 ms, *SEM* = 20; *t*(30) = -3.907, *p* < .014).

Results for interaction between valence and subjective significance within each level of arousal are presented in Fig. B1, middle row.

Thirdly, analyses within each level of subjective significance revealed statistically significant interaction effects related to the levels of valence and arousal in low (*F*(4, 120) = 8.591, *p* < .001), medium (*F*(4, 120) = 5.000, *p* < .001), and in high significance (*F*(4, 120) = 6.082, *p* < .001). Post-hoc test significant effects have previously been described. Results for interaction between valence and arousal within each level of subjective significance are presented in Fig. B1, bottom row.

##### *Figure B1. Interaction between valence, arousal, and significance for reaction times. Top row: within each level of valence interaction between arousal and subjective significance; middle row: within each level of arousal interaction between valence and subjective significance; bottom row: within each level of subjective significance interaction between valence and arousal. Vertical axis in ms. Error bars mark SEM. Horizontal lines with asterixis mark significant differences (* p <.05, ** p< .01, *** p< .001).*


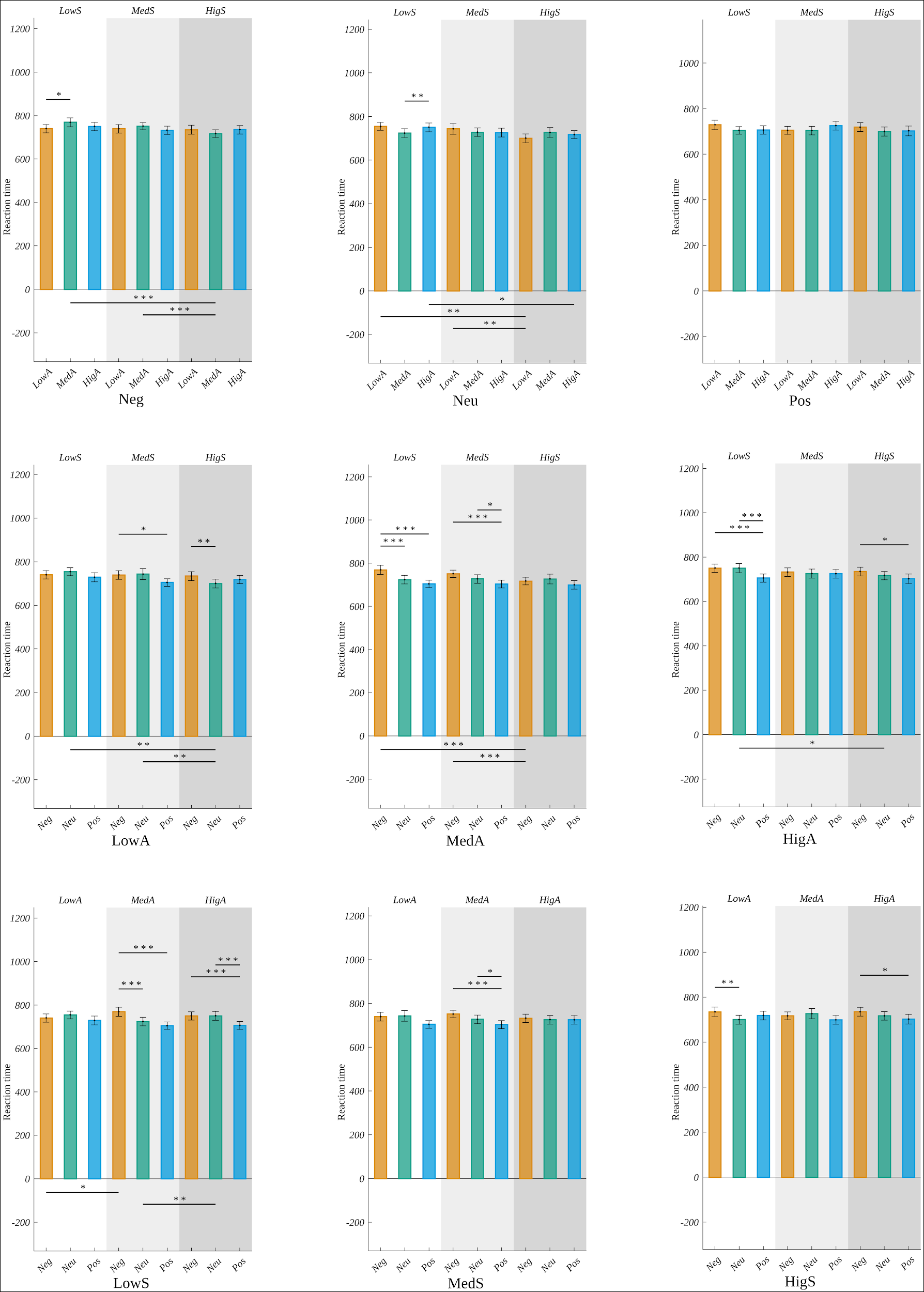


### **Appendix 3C. Detailed results of tests of amplitude related to words and pseudowords in the 265 – 400 ms time-window.**

Table C1. Detailed results of post-hoc tests of the differences between ERPs amplitude for words and pseudowords in each of the ROIs. Time-window 265-400ms (****p< .001).*

|  | Word | | | Pseudoword | | |  |
| --- | --- | --- | --- | --- | --- | --- | --- |
|  | *M (SEM)* | *MIN* | *MAX* | *M (SEM)* | *MIN* | *MAX* | *t*(30) |
| LF | 3.49 (0.46) | -0.76 | 8.30 | 1.81 (0.36) | -1.17 | 6.12 | 9.019*** |
| CF | 2.72 (0.64) | -3.72 | 10.29 | 0.97 (0.51) | -4.65 | 6.31 | 8.891*** |
| RF | 3.46 (0.53) | -1.83 | 9.91 | 1.79 (0.43) | -2.83 | 6.38 | 9.556*** |
| LP | 1.59 (0.46) | -3.14 | 6.87 | 0.65 (0.41) | -4.35 | 5.44 | 6.481*** |
| RP | 2.68 (0.44) | -1.15 | 7.42 | 1.64 (0.39) | -1.62 | 6.49 | 7.403*** |
